# Underweight, Markers of Cachexia, and Mortality in Acute Myocardial Infarction: A Prospective Cohort Study of Elderly Medicare Beneficiaries

**DOI:** 10.1371/journal.pmed.1001998

**Published:** 2016-04-19

**Authors:** Emily M. Bucholz, Hannah A Krumholz, Harlan M. Krumholz

**Affiliations:** 1 Department of Pediatrics, Boston Children’s Hospital and Boston Medical Center, Boston, Massachusetts, United States of America; 2 Yale School of Medicine and Yale School of Public Health, New Haven, Connecticut, United States of America; 3 Yale College, New Haven, Connecticut, United States of America; 4 Section of Cardiovascular Medicine, Department of Internal Medicine, Yale School of Medicine, New Haven, Connecticut, United States of America; 5 Center for Outcomes Research and Evaluation, Yale–New Haven Hospital, New Haven, Connecticut, United States of America; 6 Robert Wood Johnson Foundation Clinical Scholars Program, Department of Medicine, Yale School of Medicine, New Haven, Connecticut, United States of America; 7 Department of Health Policy and Management, Yale School of Public Health, New Haven, Connecticut, United States of America; Stanford University, UNITED STATES

## Abstract

**Background:**

Underweight patients are at higher risk of death after acute myocardial infarction (AMI) than normal weight patients; however, it is unclear whether this relationship is explained by confounding due to cachexia or other factors associated with low body mass index (BMI). This study aimed to answer two questions: (1) does comprehensive risk adjustment for comorbid illness and frailty measures explain the higher mortality after AMI in underweight patients, and (2) is the relationship between underweight and mortality also observed in patients with AMI who are otherwise without significant chronic illness and are presumably free of cachexia?

**Methods and Findings:**

We analyzed data from the Cooperative Cardiovascular Project, a cohort-based study of Medicare beneficiaries hospitalized for AMI between January 1994 and February 1996 with 17 y of follow-up and detailed clinical information to compare short- and long-term mortality in underweight and normal weight patients (*n* = 57,574). We used Cox proportional hazards regression to investigate the association of low BMI with 30-d, 1-y, 5-y, and 17-y mortality after AMI while adjusting for patient comorbidities, frailty measures, and laboratory markers of nutritional status. We also repeated the analyses in a subset of patients without significant comorbidity or frailty.

Of the 57,574 patients with AMI included in this cohort, 5,678 (9.8%) were underweight and 51,896 (90.2%) were normal weight at baseline. Underweight patients were older, on average, than normal weight patients and had a higher prevalence of most comorbidities and measures of frailty. Crude mortality was significantly higher for underweight patients than normal weight patients at 30 d (25.2% versus 16.4%, *p <* 0.001), 1 y (51.3% versus 33.8%, *p <* 0.001), 5 y (79.2% versus 59.4%, *p <* 0.001), and 17 y (98.3% versus 94.0%, *p <* 0.001). After adjustment, underweight patients had a 13% higher risk of 30-d death and a 26% higher risk of 17-y death than normal weight patients (30-d hazard ratio [HR] 1.13, 95% CI 1.07–1.20; 17-y HR 1.26, 95% CI 1.23–1.30). Survival curves for underweight and normal weight patients separated early and remained separate over 17 y, suggesting that underweight patients remained at a significant survival disadvantage over time. Similar findings were observed among the subset of patients without comorbidity at baseline. Underweight patients without comorbidity had a 30-d adjusted mortality similar to that of normal weight patients but a 21% higher risk of death over the long term (30-d HR 1.08, 95% CI 0.93–1.26; 17-y HR 1.21, 95% CI 1.14–1.29). The adverse effects of low BMI were greatest in patients with very low BMIs. The major limitation of this study was the use of surrogate markers of frailty and comorbid conditions to identify patients at highest risk for cachexia rather than clear diagnostic criteria for cachexia.

**Conclusions:**

Underweight BMI is an important risk factor for mortality after AMI, independent of confounding by comorbidities, frailty measures, and laboratory markers of nutritional status. Strategies to promote weight gain in underweight patients after AMI are worthy of testing.

## Abstract 2

In this prospective cohort study of elderly medicare beneficiaries, Harlan Krumholz and colleagues investigate the association between underweight and mortality after acute myocardial infarction independent of cachexia and frailty.

## Editors' Summary

**Background:**

A heart attack, or acute myocardial infarction (AMI), is a potentially fatal medical emergency that occurs when part of the heart muscle dies because the blood supply to the heart becomes blocked, usually by a blood clot. Every year in the US alone, more than three-quarters of a million people have a heart attack—more than one person every minute. Heart attacks are usually caused by coronary artery disease. With age, fatty deposits (atherosclerotic plaque) coat the walls of arteries, the vessels that supply the organs of the body with oxygen and nutrients. Coronary artery disease develops when plaques form in the arteries that supply the heart. A heart attack occurs when a blood clot forms in the narrowed vessel or when a plaque ruptures and triggers clot formation. Symptoms of a heart attack include chest pain, shortness of breath, and feeling lightheaded. Treatments for AMI include dissolving the blood clot with drugs and surgically opening up or bypassing the blocked artery.

**Why Was This Study Done?:**

Underweight people are at a higher risk of death after AMI than normal weight people, but is being underweight a direct risk factor for death after AMI? “Confounding” by cachexia—unintentional weight loss, muscle wasting, and fatigue that occur in the setting of chronic disease—could explain excess mortality (death) among underweight patients. That is, people who are underweight may have a higher risk of death post-AMI than normal weight people because they have another underlying disease that has caused them to lose weight. If the relationship between being underweight and post-AMI mortality is largely explained by a comorbid (coexisting) illness, managing this underlying condition may improve outcomes, whereas if being underweight is an independent risk factor for death after AMI, promoting weight gain may improve outcomes. In this prospective cohort study, the researchers investigate whether comprehensive risk adjustment for comorbid illness and frailty measures explains the higher mortality after AMI in underweight patients, and they ask whether the relationship between being underweight and mortality is also observed post-AMI in patients who have no other significant chronic illness.

**What Did the Researchers Do and Find?:**

The researchers used data from the Cooperative Cardiovascular Project, a US quality improvement initiative in which a cohort (group) of Medicare beneficiaries hospitalized for AMI were followed for many years (Medicare is a government-run program that funds healthcare for people aged ≥65 years in the US). Specifically, they analyzed short- and long-term mortality among 57,574 underweight and normal weight patients (individuals with a body mass index [BMI] of <18.5 kg/m^2^ and 18.5–24.9 kg/m^2^, respectively; BMI is an indicator of body fat calculated by dividing a person’s weight in kilograms by their height in meters squared). Crude mortality (deaths from all causes without adjustment for other factors likely to affect the risk of death) was higher among underweight patients than among normal weight patients at 30 days and 1, 5, and 17 years after AMI. After adjustment for comorbidities that cause cachexia (for example, cancer and chronic liver disease), variables reflecting frailty (such as mobility), and two laboratory measures of nutritional status, underweight patients had a 13% higher risk of death at 30 days and a 26% higher risk of death over 17 years than normal weight patients. Notably, among patients without comorbidity, underweight patients had a 21% higher risk of death over 17 years than normal weight patients.

**What Do These Findings Mean?:**

These findings suggest that, although adjustment for markers of cachexia attenuated some of the excess risk of death among underweight patients compared to normal weight patients, being underweight is an important independent risk factor for death after AMI. Moreover, they suggest that underweight patients have a survival disadvantage compared with normal weight patients for many years after AMI. Because the researchers had no information on whether patients had recently lost weight (a more direct indicator of cachexia), they relied on markers of frailty and nutritional status and the presence of comorbidities to identify the patients at highest risk of cachexia, which may limit the accuracy of these findings. Importantly, however, these findings highlight the need for further research on underweight patients who have coronary artery disease and suggest that it may be worth testing strategies to promote weight gain in underweight patients after AMI as a way to reduce the excess mortality in this group of patients compared to normal weight patients.

**Additional Information:**

This list of resources contains links that can be accessed when viewing the PDF on a device or via the online version of the article at http://dx.doi.org/10.1371/journal.pmed.1001998.

- The US Centers for Disease Control and Prevention has detailed information on coronary artery disease and myocardial infarction
- The NIH Senior Health website includes information on heart attack
- The UK National Health Service Choices website provides information about all aspects of coronary artery disease and heart attack (including personal stories) and information about being underweight
- The American Heart Association provides information on all aspects of cardiovascular disease and tips on keeping the heart healthy; its website includes personal stories about heart attack
- The US National Heart, Lung, and Blood Institute also provides information on coronary artery disease and heart attack (in English and Spanish)
- MedlinePlus provides links to other sources of information on heart attack and coronary artery disease (in English and Spanish)
- Wikipedia has a page on cachexia (note that Wikipedia is a free online encyclopedia that anyone can edit; available in several languages)

## Introduction

Underweight patients are at significantly higher risk of death after acute myocardial infarction (AMI) than patients with weights in the normal range [1–8]. Prior studies have largely attributed the excess mortality in underweight patients to confounding by cachexia, defined as unintentional weight loss, muscle atrophy, and fatigue that occur in the setting of chronic disease [3,9–12]; however, most studies lack information on measures of cachexia and thus are unable to test this hypothesis. As a result, it is unclear whether low body mass index (BMI) is a marker of generalized illness and risk or represents an independent risk factor worthy of attention in its own right.

Cachexia is likely mediated by neuroendocrine, metabolic, and inflammatory pathways [13,14], and the criteria used to define cachexia vary widely. Definitions in studies usually include some combination of current BMI or recent weight loss, symptoms of fatigue or anorexia, and biochemical markers in the setting of chronic disease [15]. In the absence of information on recent weight trends and patient-reported symptoms, studies in non-AMI populations have used other markers of severe illness or frailty as proxies to determine which patients are likely cachectic [16–20]. However, most studies in AMI populations lack information on patient comorbidities, frailty measures, or laboratory markers to identify these patients.

Understanding how low BMI relates to post-AMI mortality has implications for the care and management of underweight patients in hospital and after discharge. Because nutritional supplementation alone is often ineffective in reversing cachexia, treatment focuses instead on managing underlying conditions [21,22]. If low BMI is associated with mortality after AMI independent of other conditions and function, then promoting weight gain and optimizing caloric intake in underweight patients after AMI may improve outcomes, a hypothesis that could be tested. However, if the relationship between underweight and post-AMI mortality is largely explained by cachexia or other comorbid illnesses, then managing the underlying condition is as important as improving nutritional status alone.

Accordingly, we sought to further delineate the relationship between low BMI, cachexia, and mortality after AMI. We used detailed chart-abstracted data from a large cohort of Medicare beneficiaries with AMI to compare short- and long-term mortality in underweight and normal weight patients while adjusting for numerous patient comorbidities, frailty measures, and laboratory markers of nutritional status. In addition we repeated the analyses in a subset of patients without significant chronic illness. We posed two questions: (1) does comprehensive risk adjustment for comorbid illness and frailty measures explain the higher mortality after AMI in underweight patients, and (2) is the relationship between underweight and mortality also observed in patients with AMI who are otherwise without significant chronic illness and are presumably free of cachexia? Finally, we examined interactions of sex and age with underweight to determine whether the effect of underweight varies by other patient characteristics.

## Methods

### Study Sample

We analyzed data from the Cooperative Cardiovascular Project, a quality improvement initiative designed by the Centers for Medicare and Medicaid Services to evaluate the quality of care delivered to patients with AMI in the US [23,24]. In brief, the Cooperative Cardiovascular Project sampled fee-for-service Medicare beneficiaries hospitalized with a principal discharge diagnosis code of AMI (International Classification of Diseases, Ninth Revision, Clinical Modification code 410) from acute-care nongovernmental hospitals in the US between January 1994 and February 1996. Trained personnel performed the detailed medical record abstraction using an automated system to ensure standardization of techniques. Data quality was monitored by random reabstractions and assessment of reliability statistics. This study was approved by the Yale University institutional review board (Protocol HIC #1209010804). Informed consent was not required for this study by the Yale University institutional review board because all data had been previously collected in 1994–1996 through a centralized Medicare initiative. All data were deidentified during the analytic stages.

For this study, we limited our analysis to patients ≥65 y old who were hospitalized with AMI that was confirmed by medical record. The diagnosis of AMI was confirmed by elevated cardiac enzymes (e.g., elevation of creatine kinase–myocardial band level [>5% of total creatine kinase] or elevation of lactate dehydrogenase enzyme [LDH] level with isoenzyme reversal [LDH_1_ > LDH_2_]) or the presence of at least two of the following: chest pain, 2-fold elevation in total creatine kinase, or diagnostic changes on electrocardiogram (e.g., ST-segment elevation or new pathological Q waves). If patients were admitted more than once for AMI during the study period, we included only the first admission. Finally, we excluded patients with missing height (*n* = 24,014) or weight (*n* = 13,180) data because we could not calculate BMI for these patients.

### Variable Definitions

BMI values were calculated from patients’ chart-documented height and weight at the time of AMI hospitalization. We used criteria from the Centers for Disease Control and Prevention to classify patients as underweight (BMI < 18.5 kg/m^2^) or normal weight (18.5 kg/m^2^ ≤ BMI < 25 kg/m^2^).

The primary outcomes were mortality at 30 d, 1 y, 5 y, and 17 y calculated from the day of hospital admission. Vital status was ascertained over 17 y through linkage to the 1994–2012 Medicare Denominator Files, which provide complete death information on all beneficiaries enrolled in Medicare.

Cachexia-related variables were identified using prior literature, clinical judgment, and face validity for their association with underweight, cachexia, and frailty. Specifically, we included comorbidities that are known to cause cachexia (i.e., congestive heart failure [CHF], chronic obstructive pulmonary disease [COPD], cerebrovascular accident (CVA) or stroke, cirrhosis/liver disease, chronic kidney disease [CKD], infection with human immunodeficiency virus [HIV] or immunocompromised state, cancer, Alzheimer disease or dementia, and other terminal illnesses). Comorbidities were ascertained through chart-documented medical history information, which was part of the patient’s medical record or collected during the index admission. In addition, we included two laboratory markers of nutritional status extracted from patient charts (anemia [hematocrit < 30%] and hypoalbuminemia [serum albumin < 3 g/dl]), and three variables reflecting frailty prior to admission (admission from a skilled nursing facility [SNF], mobility, and urinary continence on admission). Mobility (walks independently, walks with assistance, unable to walk) and incontinence (continent, totally/occasionally incontinent, anuric) on admission were determined from provider notes and chart-documented impairments. We selected these variables because validated frailty scales have typically included some combination of activities of daily living or self-sufficiency [25–27], urinary continence [26–28], mobility [25,28–30], stamina [25,28], and cognitive functioning [27–30]. Although we lacked information on cognitive functioning and stamina, we incorporated assessments of mobility and continence, and we used residence at a SNF as a proxy for self-sufficiency.

In addition to cachexia-related variables, we included information on patient demographics (age, gender, race), cardiovascular risk factors (diabetes, hypertension, smoking, prior coronary artery disease [CAD]), clinical presentation (Killip classification, systolic blood pressure, heart rate on presentation, ST-elevation AMI, anterior infarction, cardiac arrest on admission, renal insufficiency), treatment (percutaneous coronary intervention [PCI] or coronary artery bypass grafting [CABG] within the first 30 d, fibrinolytic therapy, aspirin on admission, and beta-blockers on admission), and in-hospital complications. Patients with missing systolic blood pressure were assigned the median value in the overall cohort and a dummy variable to denote missing. Patients with missing categorical variables (mobility, urinary continence, and PCI/CABG) were included in the model using dummy variables for missing data.

### Statistical Analyses

Baseline characteristics (i.e., at the index admission) were compared between underweight and normal weight patients using chi-squared tests for categorical variables and Student’s *t* tests for continuous variables. To evaluate the relationship of underweight to short- and long-term mortality, we performed two sets of analyses modeling BMI first as a categorical and then as a continuous variable. In analyses of BMI as a categorical variable, we used chi-squared tests, Kaplan–Meier curves with log-rank tests, and Cox proportional hazards regression to compare unadjusted and adjusted mortality at 30 d, 1 y, 5 y, and 17 y after AMI between normal weight and underweight patients. In addition, we calculated conditional hazard ratios (HRs) for the intervals 0 to 30 d, >30 d to 1 y, >1 to 5 y, and >5 to 17 y to determine whether underweight patients were at higher risk of death early after AMI or accrued a survival disadvantage over time. Interaction terms for sex and age with underweight were tested in all models.

In the second set of analyses, we modeled BMI as a continuous variable to better characterize the shape of the association of low BMI with 1- and 17-y mortality. Specifically, we modeled the hazards of death relative to patients with a BMI of 20 kg/m^2^ using proportional hazards regression restricted cubic spline models with knots located at each BMI integer value [31,32]. This approach combines linear and nonlinear transformations of BMI at different sections of the BMI curve to identify the best-fitting transformations for the association between BMI and mortality. Models were then repeated adjusting for the same covariates above.

Finally, because multivariate adjustment may be insufficient to remove confounding by cachexia, we repeated the above analyses in a subset of patients without significant comorbidity or frailty (*n* = 20,587). Specifically, we excluded patients with CHF, COPD, CVA/stroke, cirrhosis/liver disease, CKD, HIV, cancer, Alzheimer disease/dementia, terminal illness, anemia, or hypoalbuminemia; patients admitted from SNFs; and patients with mobility issues or incontinence. All statistical analyses were performed using SAS 9.2 (SAS Institute).

## Results

Our sample included 5,678 (9.8%) underweight patients and 51,896 (90.2%) normal weight patients. Underweight and normal weight patients represented 44% of all eligible patients. Compared with patients with recorded BMI values, patients with missing BMI were on average older (mean age 78.6 versus 76.0 y, *p <* 0.001). In addition, they were more likely to be admitted from SNFs (14.0% versus 5.0%, *p <* 0.001) and less likely to be mobile (68.1% versus 80.7%, *p <* 0.001) or continent (82.0% versus 92.0%, *p <* 0.001) on admission. Patients with missing BMI had higher in-hospital and 17-y mortality rates (in hospital: 27.0% versus 11.9%, *p <* 0.001; 17 y: 96.1% versus 92.3%, *p <* 0.001).

Underweight patients, compared with normal weight patients, were older, on average, and a greater percentage of them were women (Table 1). Although underweight patients had a lower prevalence of diabetes, hypertension, and prior CAD, they had significantly higher rates of smoking, nearly all other comorbidities (including CHF, COPD, CVA/stroke, CKD, cancer, and Alzheimer disease/dementia), anemia, and hypoalbuminemia. They were also more likely to be admitted from SNFs and to have decreased mobility and urinary continence on admission (Table 1). Underweight patients were significantly less likely to receive guideline-based therapies on admission including aspirin, beta-blockers, fibrinolytic therapy, and revascularization procedures.

**Table 1:** Baseline characteristics, clinical presentation, in-hospital events, and mortality for underweight versus normal weight patients.

Category	Characteristic	All Patients (*n =* 57,574)			Subset of Patients without Significant Comorbidity (*n =* 20,587)		
		Underweight (*n =* 5678)	Normal Weight (*n =* 51,896)	*p*-Value	Underweight (*n =* 1081)	Normal Weight (*n =* 19,506)	*p*-Value
**Demographics**	**Age**	80.3 (7.9)	77.7 (7.4)	<0.001	78.7 (7.9)	75.9 (6.9)	<0.001
	**Female**	3,847 (67.8%)	25,411 (49.0%)	<0.001	748 (69.2%)	9,027 (46.3%)	<0.001
	**Nonwhite race**	568 (10.0%)	4,603 (8.9%)	0.006	110 (10.2%)	1,588 (8.1%)	0.018
**Cardiovascular risk factors**	**Diabetes mellitus**	897 (15.8%)	12,562 (24.2%)	<0.001	140 (13.0%)	3,666 (18.8%)	<0.001
	**Hypertension**	3,073 (54.1%)	30,051 (57.9%)	<0.001	577 (53.4%)	10,232 (52.5%)	0.555
	**Smoker**	1,213 (21.4%)	8,839 (17.0%)	<0.001	219 (20.3%)	3,176 (16.3%)	0.001
	**Prior CAD**	563 (9.9%)	8,794 (17.0%)	<0.001	114 (10.6%)	3,411 (17.5%)	<0.001
**Comorbidities**	**CHF**	1,689 (29.8%)	11,425 (22.0%)	<0.001	—	—	—
	**COPD**	1,997 (35.2%)	11,353 (21.9%)	<0.001	—	—	—
	**CVA/stroke**	930 (16.4%)	7,502 (14.5%)	<0.001	—	—	—
	**Cirrhosis/liver disease**	31 (0.6%)	197 (0.4%)	0.058	—	—	—
	**CKD**	339 (6.0%)	2,643 (5.1%)	0.005	—	—	—
	**HIV/immunocompromised**	113 (2.0%)	932 (1.8%)	0.298	—	—	—
	**Cancer**	201 (3.5%)	1,469 (2.8%)	0.003	—	—	—
	**Alzheimer disease/dementia**	712 (12.5%)	3,297 (6.4%)	<0.001	—	—	—
	**Terminal illness** *	47 (0.8%)	176 (0.3%)	<0.001	—	—	—
**Markers of nutritional status**	**Anemia (hematocrit < 30%)**	645 (11.4%)	4,115 (7.9%)	<0.001	—	—	—
	**Hypoalbuminemia (serum albumin < 3 g/dl)**	521 (9.2%)	2,504 (4.8%)	<0.001	—	—	—
**Measures of frailty**	**Admitted from SNF**	788 (13.9%)	3,342 (6.4%)	<0.001	—	—	—
	**Mobility at admission**			<0.001	—	—	—
	Walks independently	3,675 (64.7%)	41,053 (79.1%)		—	—	—
	Walks with assistance	1,427 (25.2%)	8,298 (16.0%)		—	—	—
	Unable to walk	388 (6.8%)	1,422 (2.7%)		—	—	—
	Missing data	188 (3.3%)	1,123 (2.2%)		—	—	—
	**Urinary continence at admission**			<0.001	—	—	—
	Continent	4,760 (83.8%)	47,221 (91.0%)		—	—	—
	Totally/occasionally incontinent	720 (12.7%)	3,485 (6.7%)		—	—	—
	Anuric	35 (0.6%)	256 (0.5%)		—	—	—
	Missing data	163 (2.9%)	934 (1.8%)		—	—	—
**Clinical presentation**	**Killip classification > 2**	2,274 (40.1%)	18,958 (36.5%)	<0.001	285 (26.4%)	4,298 (22.0%)	0.001
	**SBP (mm Hg)**	137 (34)	143 (32)	<0.001	142 (32)	145 (31)	0.002
	**Missing data on SBP**	31	206	<0.001	4	57	0.002
	**Heart rate (bpm)**	93 (26)	88 (25)	<0.001	85 (24)	82 (23)	<0.001
	**STEMI**	1,675 (29.5%)	15,205 (29.3%)	0.752	369 (34.1%)	6,373 (32.7%)	0.318
	**Anterior infarction**	2,877 (50.7%)	24,802 (47.8%)	<0.001	566 (52.4%)	9,340 (47.9%)	0.004
	**Cardiac arrest on admission**	170 (3.0%)	1,572 (3.0%)	0.883	22 (2.0%)	453 (2.3%)	0.540
	**Renal insufficiency**	1,092 (19.3%)	6,927 (13.4%)	<0.001	84 (7.8%)	826 (4.2%)	<0.001
**Treatment**	**PCI/CABG in first 30 d**	661 (11.6%)	13,984 (27.0%)	<0.001	277 (25.6%)	7,793 (40.0%)	<0.001
	**Missing data on PCI/CABG in first 30 d**	176 (3.1%)	1,574 (3.0%)		23 (2.1%)	546 (2.8%)	
	**Fibrinolytic therapy**	548 (9.7%)	8,533 (16.4%)	<0.001	219 (20.3%)	4,978 (25.5%)	<0.001
	**Aspirin on admission for eligible patients**	2,629/4,098 (64.2%)	29,337/38,897 (75.4%)	<0.001	696/907 (76.7%)	13,755/16,712 (82.3%)	<0.001
	**Beta-blockers on admission for eligible patients**	824/1,756 (46.9%)	13,797/23,510 (58.7%)	<0.001	431/792 (54.4%)	9,300/15,010 (62.0%)	<0.001
**In-hospital complications**	**Cardiac arrest within 48 h**	170 (3.0%)	1,572 (3.0%)	0.883	22 (2.0%)	453 (2.3%)	0.540
	**Atrial fibrillation/flutter**	1,416 (24.9%)	10,951 (21.1%)	<0.001	230 (21.3%)	3,160 (16.2%)	<0.001
	**Bleeding/hemorrhage**	977 (17.2%)	9,031 (17.4%)	0.712	170 (15.7%)	3,130 (16.1%)	0.780
	**CVA**	209 (3.7%)	1,588 (3.1%)	0.030	29 (2.7%)	423 (2.2%)	0.383
	**Missing data on CVA**	5 (0.1%)	33 (0.1%)		0 (0%)	12 (0.1%)	
	**Reinfarction**	198 (3.5%)	1,850 (3.6%)	0.873	49 (4.5%)	727 (3.7%)	0.155
	**Missing data on reinfarction**	185 (3.3%)	1,745 (3.4%)		27 (2.5%)	641 (3.3%)	
	**Shock**	444 (7.8%)	3,491 (6.7%)	0.002	73 (6.8%)	1,047 (5.4%)	0.051
	**CHF/pulmonary edema**	2,708 (47.7%)	22,467 (43.3%)	<0.001	351 (32.5%)	5,516 (28.3%)	0.003
	**Transferred to ICU**	328 (5.8%)	3,089 (6.0%)	0.353	42 (3.9%)	973 (5.0%)	0.178
	**Missing data on transfer to ICU**	7 (0.1%)	37 (0.1%)		0 (0%)	14 (0.1%)	
	**Required intubation**	600 (10.6%)	5,539 (10.7%)	0.884	99 (9.2%)	1,567 (8.0%)	0.167
	**Missing data on intubation**	185 (3.3%)	1,745 (3.4%)		27 (2.5%)	641 (3.3%)	
**Crude mortality rates**	**In hospital**	1,212 (21.4%)	7,219 (13.9%)	<0.001	147 (13.6%)	1,680 (8.6%)	<0.001
	**30 d**	1,430 (25.2%)	8,500 (16.4%)	<0.001	180 (16.7%)	2,089 (10.7%)	<0.001
	**1 y**	2,915 (51.3%)	17,523 (33.8%)	<0.001	323 (29.9%)	3,704 (19.0%)	<0.001
	**5 y**	4,494 (79.2%)	30,841 (59.4%)	<0.001	581 (53.8%)	7,282 (37.3%)	<0.001
	**17 y**	5,581 (98.3%)	48,770 (94.0%)	<0.001	1,022 (94.5%)	17,099 (87.7%)	<0.001

Data are presented as number (percent) or mean (standard deviation).

*Terminal illness was defined as having an estimated survival less than 6 mo as determined by the treating physician.

bpm, beats per minute; ICU, intensive care unit; SBP, systolic blood pressure; STEMI, ST-elevation myocardial infarction.

**Fig 1:**
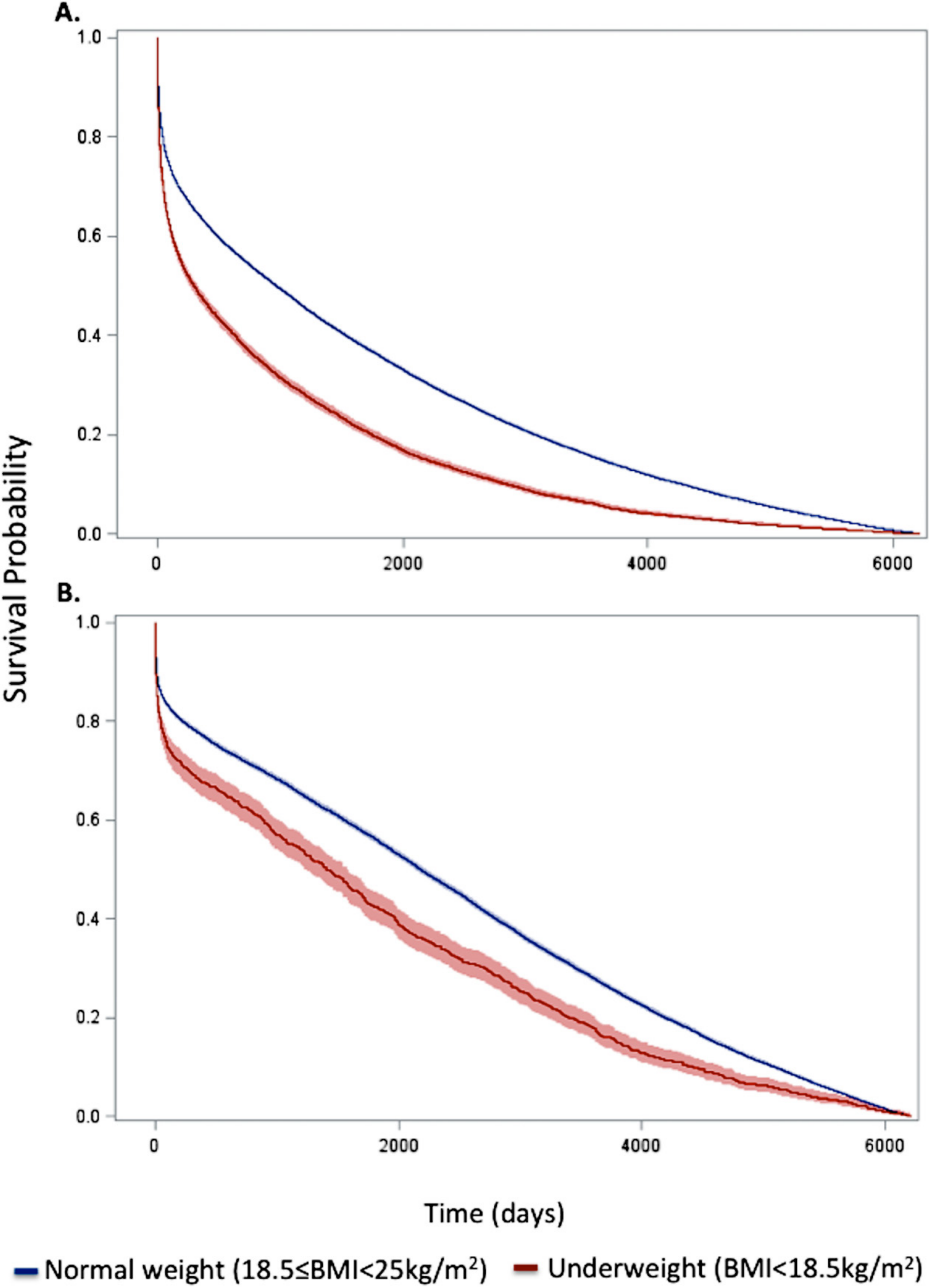
Kaplan–Meier survival curves for underweight and normal weight among all patients and the subset of patients without significant comorbidity or frailty. Curves for (A) all patients and (B) the subset of patients without significant comorbidity or frailty. The lines represent the Kaplan–Meier survivor functions, and the shaded areas are the 95% confidence limits. *p*-Value for log-rank test < 0.001 for both comparisons.

In-hospital mortality was significantly higher for underweight patients compared with normal weight patients; however, rates of most other in-hospital complications were similar (Table 1). Crude mortality was significantly higher for underweight patients than normal weight patients at 30 d (25.2% versus 16.4%), 1 y (51.3% versus 33.8%), 5 y (79.2% versus 59.4%), and 17 y (98.3% versus 94.0%) (all *p <* 0.001) (Fig 1; Table 1). Conditional HRs showed divergence of the survival curves over all 17 y of follow-up, suggesting that underweight patients remained at a significant survival disadvantage over time (Table 2). After adjustment for patient and treatment characteristics during the index admission, underweight patients remained at a significant survival disadvantage across all follow-up time points: the curves diverged early and remained separate over the 17 y of follow-up. Underweight patients had a 13% greater risk of death within the first 30 d and a 26% greater risk of death over the full 17 y of follow-up (30-d adjusted HR 1.13, 95% CI 1.07–1.20; 17-y adjusted HR 1.26, 95% CI 1.23–1.30) (Table 2).

**Table 2:** Short- and long-term overall and conditional hazard ratios for underweight versus normal weight (reference) patients.

Analysis	All Patients		Subset of Patients without Significant Comorbidity	
	Unadjusted Underweight HR (95% CI)	Adjusted* Underweight HR (95% CI)	Unadjusted Underweight HR (95% CI)	Adjusted* Underweight HR (95% CI)
**Overall HR**				
30 d	1.61 (1.52, 1.70)	1.13 (1.07, 1.20)	1.60 (1.37, 1.86)	1.08 (0.93, 1.26)
1 y	1.72 (1.66, 1.79)	1.25 (1.20, 1.30)	1.67 (1.50, 1.88)	1.18 (1.05, 1.32)
5 y	1.73 (1.67, 1.78)	1.27 (1.23, 1.31)	1.64 (1.51, 1.79)	1.22 (1.12, 1.33)
17 y	1.67 (1.62, 1.71)	1.26 (1.23, 1.30)	1.51 (1.42, 1.61)	1.21 (1.14, 1.29)
**Conditional HR**				
0 to 30 d	1.61 (1.52, 1.70)	1.13 (1.07, 1.20)	1.60 (1.37, 1.86)	1.08 (0.93, 1.26)
>30 d to 1 y	1.84 (1.75, 1.95)	1.38 (1.30, 1.46)	1.79 (1.51, 2.12)	1.34 (1.12, 1.59)
>1 y to 5 y	1.73 (1.65, 1.83)	1.31 (1.25, 1.39)	1.60 (1.41, 1.81)	1.28 (1.12, 1.45)
>5 y to 17 y	1.45 (1.37, 1.54)	1.20 (1.13, 1.28)	1.37 (1.25, 1.51)	1.18 (1.07, 1.30)

HRs compare hazards of death in underweight patients versus normal weight patients.

*Multivariable analyses were adjusted for patient demographics (age, sex, race), cardiovascular risk factors (diabetes, hypertension, smoking, prior CAD), comorbidities (CHF, COPD, CVA/stroke, cirrhosis/liver disease, CKD, HIV or immunocompromised state, cancer, Alzheimer disease/dementia, terminal illness), markers of nutritional status (anemia, hypoalbuminemia), measures of frailty (admission from a SNF, mobility on admission, urinary continence on admission), clinical presentation (Killip classification, systolic blood pressure, heart rate, ST-elevation AMI, anterior infarction, cardiac arrest on admission, renal insufficiency), and treatment (PCI or CABG within the first 30 d of admission, fibrinolytic therapy, aspirin on admission, and beta-blockers on admission).

**Fig 2:**
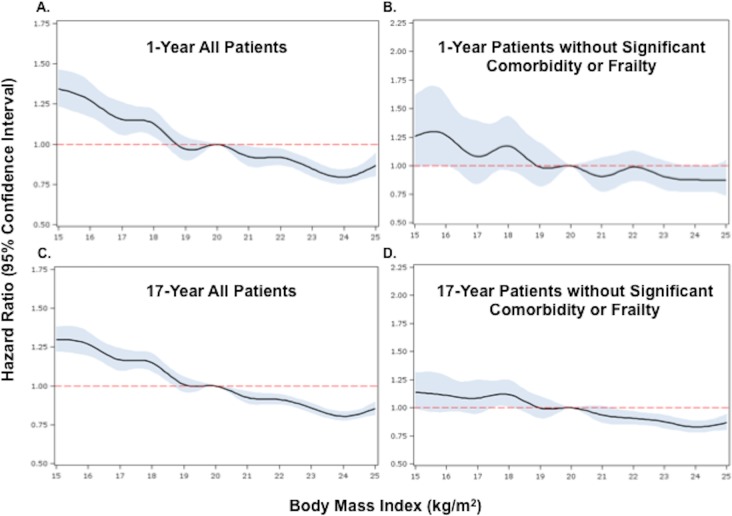
Adjusted Cox proportional hazards regression restricted cubic spline models for all patients and for the subset of patients without significant comorbidity or frailty. (A) and (B) show 1-y adjusted mortality for all patients and for the subset of patients without significant comorbidity or frailty, respectively. (C) and (D) show 17-y adjusted mortality for all patients and for patients without significant comorbidity or frailty. The reference category is patients with a BMI of 20 kg/m^2^. In each panel, the black line denotes the estimated HR, and gray shading indicates the 95% confidence limits. Unadjusted 1- and 17-y curves for all patients and for the subset of patients without significant comorbidity or frailty are shown in S1 Fig. Analyses were adjusted for patient demographics (age, sex, race), cardiovascular risk factors (diabetes, hypertension, smoking, prior CAD), comorbidities (CHF, COPD, CVA/stroke, cirrhosis/liver disease, CKD, HIV or immunocompromised state, cancer, Alzheimer disease/dementia, terminal illness), markers of nutritional status (anemia, hypoalbuminemia), measures of frailty (admission from an SNF, mobility on admission, urinary continence on admission), clinical presentation (Killip classification, systolic blood pressure, heart rate, ST-elevation AMI, anterior infarction, cardiac arrest on admission, renal insufficiency), and treatment (PCI or CABG within the first 30 d of admission, fibrinolytic therapy, aspirin on admission, and beta-blockers on admission).

When BMI was examined as a continuous variable, there was an inverse relationship between BMI and the hazards of death at both 1 and 17 y. The highest risk of death was observed in those with very low BMI (<17 kg/m^2^), and the lowest risk in those with BMIs in the upper range of normal (>24 kg/m^2^). This relationship persisted after adjustment (Figs 2 and S1).

To further reduce the potential for confounding by cachexia, we repeated the analyses in a subset of underweight and normal weight patients without significant comorbidities or markers of frailty. Compared with the previous analyses, a smaller percentage of the cohort was classified as underweight (*n* = 1,081, 5.2%). However, baseline comparisons between underweight and normal weight patients in this subset were similar to those in the previous analyses (Table 1). Crude mortality rates were higher for underweight patients across all follow-up time points (30 d: 16.7% versus 10.7%; 1 y: 29.9% versus 19.0%; 5 y: 53.8% versus 37.3%; 17 y: 94.5% versus 87.7%) (Fig 1; Table 1), and risk estimates were similar to those in the analyses of all patients (Table 2). Conditional HRs again showed early divergence of the survival curves, which remained separate over all 17 y of follow-up (Table 2). After adjustment, underweight and normal weight patients had a similar risk of 30-d mortality (HR 1.08, 95% CI 0.93–1.26); however, the long-term risk of death in underweight patients remained significantly higher than that in normal weight patients (17-y HR 1.21, 95% CI 1.14–1.29). Similarly, when BMI was modeled as a continuous variable, we observed an inverse relationship between BMI and the hazards of death; however, the magnitudes of the HRs for low BMIs were smaller in the subset of patients without significant comorbidity than in all patients (Fig 2).

**Fig 3:**
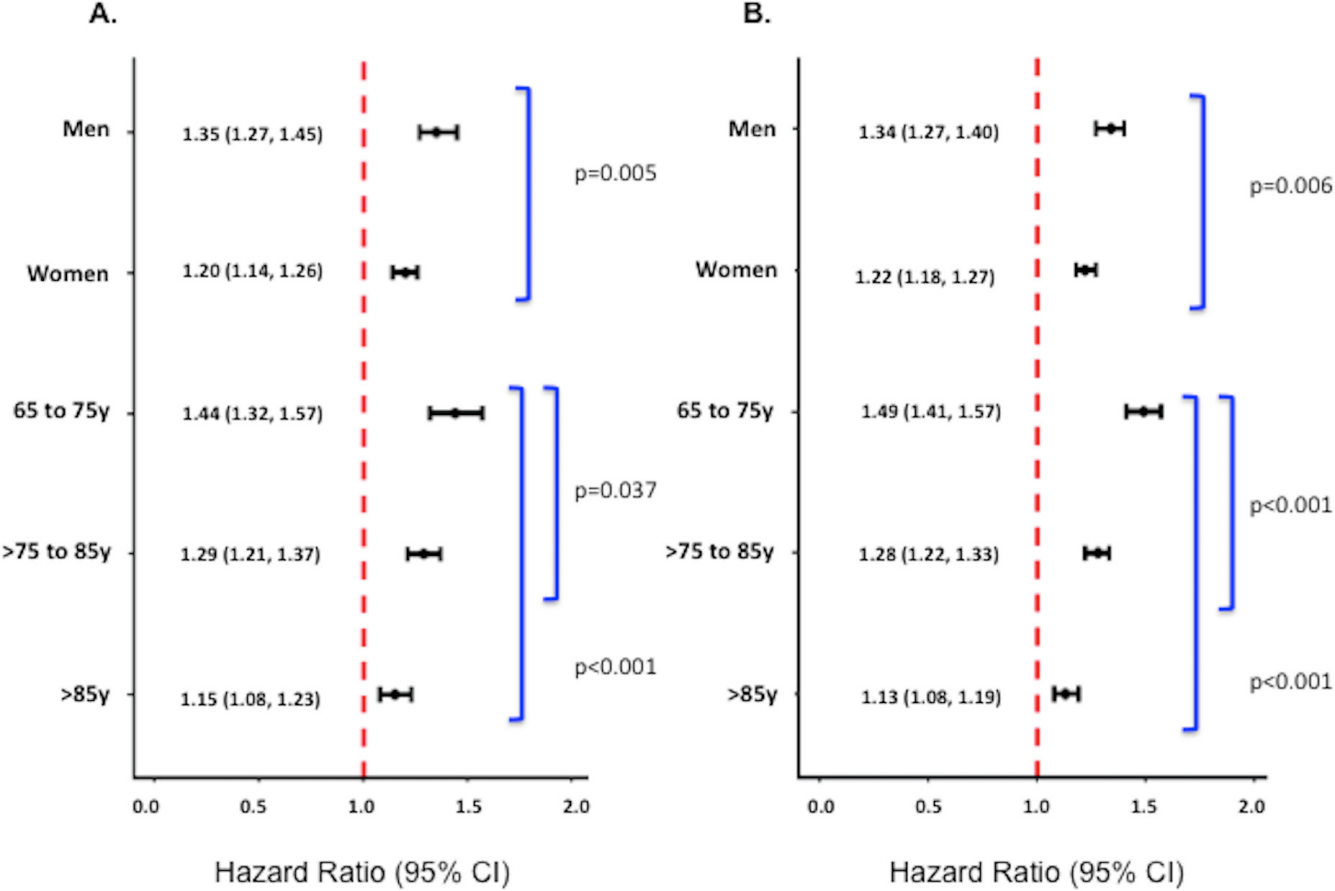
Adjusted 1-y and 17-y hazard ratios for underweight versus normal weight patients stratified by sex and age among all patients. Adjusted 1-y (A) and 17-y (B) HRs among all patients. Corresponding adjusted HRs for the subset of patients without significant comorbidity or frailty are provided in S2 Fig. Adjusted analyses were adjusted for patient demographics (age, sex, race), cardiovascular risk factors (diabetes, hypertension, smoking, prior CAD), comorbidities (CHF, COPD, CVA/stroke, cirrhosis/liver disease, CKD, HIV or immunocompromised state, cancer, Alzheimer disease/dementia, terminal illness), markers of nutritional status (anemia, hypoalbuminemia), measures of frailty (admission from an SNF, mobility on admission, urinary continence on admission), clinical presentation (Killip classification, systolic blood pressure, heart rate, ST-elevation AMI, anterior infarction, cardiac arrest on admission, renal insufficiency), and treatment (PCI or CABG within the first 30 d of admission, fibrinolytic therapy, aspirin on admission, and beta-blockers on admission).

Underweight was associated with an increased risk of death in both sexes and at all ages at both 1 and 17 y; however, the relationship between underweight and mortality was stronger in men and younger patients (65–75 y of age) (*p*-values for interactions < 0.01) (Fig 3). After limiting the cohort to patients without significant comorbidity, however, only the interaction between underweight and age on 17-y mortality was significant (S2 Fig).

## Discussion

Using detailed clinical data from a large study of elderly patients with AMI, we found that low BMI was associated with increased short- and long-term mortality after AMI. Underweight patients had a 61% to 73% higher crude risk of death than normal weight patients at all follow-up time points. The survival curves for underweight and normal weight patients diverged early and remained separate over all 17 y of follow-up, suggesting that underweight patients accrued a survival disadvantage over time. Although adjustment for markers of cachexia (comorbid conditions and measures of frailty and nutritional status) as well as other patient and treatment characteristics attenuated some of the excess risk in underweight patients, underweight patients still had a 13% to 27% higher risk of death than normal weight patients. Furthermore, when we restricted the cohort to a subset of patients without significant comorbidity or frailty, underweight patients continued to have an 8% to 22% higher risk of death than normal weight patients.

Like our study, prior studies, focusing largely on shorter-term outcomes, have consistently reported higher mortality for underweight patients [1–8]; however, these findings have been largely attributed to incomplete adjustment and confounding by other cachexia-related conditions, a conclusion that has not to our knowledge been previously tested [3,9–11]. Building on prior studies, we were able to demonstrate that although cachexia explains some of the excess mortality in underweight patients after AMI, low BMI is an independent predictor of mortality after AMI even in patients at lowest risk for cachexia. These findings point to different mechanisms in the relationship between underweight and mortality after AMI than those previously hypothesized and have implications for interventions.

Several mechanisms may explain the higher mortality in underweight patients. First, patients with low BMI have decreased physiologic reserve and fat stores, which may lower their ability to withstand insults to health over time and make them more vulnerable to adverse events. Patients with CAD have increased cardiometabolic demands due to activation of neurohormonal and inflammatory pathways [5]. Increased subcutaneous fat and energy reserves may help to overcome these catabolic changes. For underweight patients, hospitalizations for cardiac events may lead to additional weight loss, which can place them at higher risk of infection, complications, and, ultimately, repeat hospitalizations. Once in this cyclic process, patients may never fully recover to baseline and may remain at increased risk of mortality long after the index hospitalization. Indeed, our finding that the underweight and normal weight survival curves remain separated over time indicates that underweight patients are still accruing a survival disadvantage for years after the initial hospitalization.

Second, we observed that underweight patients were significantly less likely to receive guideline-recommended therapies for AMI including primary reperfusion and revascularization procedures. These lower treatment rates in underweight patients may be due to either physician bias or poorer clinical presentations on arrival. Some studies have hypothesized that the lack of functional reserve in underweight patients can lead to unfavorable hemodynamic changes during AMI, which may preclude these patients from receiving therapies [33]. Furthermore, underweight patients may be at higher risk of medication- or procedure-related complications. Prior studies have shown that underweight patients have smaller coronary vessels, which may lead to suboptimal artery-to-device ratios and higher rates of bleeding after PCI [34]. Similarly, others have proposed that medications used to treat CAD may have limited efficacy or greater toxicity in underweight patients, although there is limited evidence supporting this claim [35]. Although we adjusted for eligibility and receipt of therapies, we were unable to adjust for procedure-related variables or complications, which may offer more insight into the effectiveness of these therapies in underweight patients.

Third, the pathophysiology of AMI may be a fundamentally different process in underweight patients than in normal weight and overweight patients. Because CAD is largely attributable to the detrimental effects of adiposity and other modifiable risk factors associated with obesity, underweight patients may have an underlying genetic predisposition to CAD, which could be associated with worse prognosis [36]. Indeed, other studies have found that despite their lower prevalence of cardiovascular risk factors, underweight patients have more severe and extensive coronary disease than normal weight and overweight patients [37,38].

Finally, it is possible that residual confounding either by variables in the model or other unmeasured variables may explain the association of low BMI with mortality. Although we adjusted for and subsequently excluded patients with comorbid conditions such as CHF, COPD, and cancer, we were unable to adjust for the severity, duration, or complications of illness. Thus, it is possible that, in addition to having more comorbidity, underweight patients also had longer standing or more severe disease or were at higher risk of having undiagnosed disease, which may have compounded their risk of mortality after AMI. Additionally, we lacked information on other BMI-associated risk factors and comorbidities such as malnutrition, autoimmune and inflammatory disorders, and severe systemic illnesses or multi-organ dysfunction, which may be more prevalent in underweight patients.

To our knowledge, this is the first study to report differences in the effect of underweight on mortality after AMI by age and sex. Although the mechanisms underlying these differences are unclear, it is possible that lower BMI in men reflects a more malnourished or cachetic state since men typically have higher BMIs and lean body mass than women. We also found that underweight was more potent in younger patients. Although these differences by age may reflect our ability to detect larger differences in mortality in younger patients, who have higher overall survival, it is also possible that older age acts as an equalizer of risk because both underweight and normal weight older patients have reduced physiologic reserve to overcome acute events like AMI [39–41].

Clinically, our findings imply that underweight patients may benefit from treatment strategies that focus on promoting nutritional status and weight gain, regardless of the reason for their low BMI. Such strategies may include inpatient caloric supplementation and outpatient nutritional consults in addition to pharmacotherapy. Recently, pharmaceutical agents, such as megestrol acetate, medroxyprogesterone, ghrelin, and omega-3-fatty acid, have been used to promote weight gain and improve survival in the setting of cancer and cardiac cachexia [42–44]. Such agents may benefit underweight patients with and without cachexia after AMI; however, trials are needed to test whether use of these therapies improves weight gain in patients with AMI and whether weight gain in underweight patients improves survival after AMI. Similarly, a better understanding of why underweight patients are at increased risk of mortality after AMI—including the physiologic, therapeutic, and systems-level causes—would help us to better target therapies to improve outcomes in these patients.

Our study has some limitations. First, we were unable to directly determine which patients met the criteria for cachexia. Although many criteria exist, most include current BMI or recent weight loss, symptoms of fatigue or anorexia, and biochemical markers in the setting of chronic disease. Like many other studies, we lacked information on recent weight trends and thus relied on other markers of frailty, nutritional status, and comorbid conditions to identify patients at highest risk for cachexia. Second, many factors other than cachexia may contribute to low BMI in elderly patients, including malnutrition, sarcopenia, genetics, or increased metabolic demands. We lacked information on nutritional status and recent weight loss and therefore were unable to determine the primary cause of low BMI in underweight patients. Thus, it is possible that the effect of underweight on post-AMI mortality varies by cause. Future studies should evaluate the effect of nutritional status and lifetime changes in BMI on the relationship between underweight and mortality after AMI. Third, we used patient BMI measured at the index hospitalization. Reports from other AMI cohorts have been mixed, with some reporting minimal weight changes in the year after AMI [45] and others reporting sizeable weight gains or losses [46,47], although these studies have largely been performed in cohorts of heavier patients. Fourth, we excluded 27,690 patients (17.5% of the initial sample) for missing BMI data. Because patients with missing BMI data had higher short- and long-term mortality rates than patients in our sample, our cohort may be healthier than the general AMI population. Fifth, we lacked information on cause of death and thus could not identify the cause of the excess deaths. Finally, we used dummy variables for missing data rather than imputing missing values. Although this approach is not preferred because patients with missing data can have dissimilar values [48], we chose this approach due to the high computational cost of multiple imputations and the low missing data rates.

### Conclusions

In summary, we found that low BMI was associated with short- and long-term mortality after AMI independent of confounding by factors associated with cachexia. These findings suggest a different mechanism than previously hypothesized and highlight the need for additional research in underweight patients, who are frequently excluded from studies evaluating BMI in patients with CAD. Clinically, these findings suggest that strategies to promote weight gain in underweight patients after AMI are worthy of testing.

## Supporting Information

S1 STROBE checklist (DOCX)

S1 Fig Unadjusted Cox proportional hazards regression restricted cubic spline models for all patients and for patients without significant comorbidity or frailty. (A) and (B) show 1-y unadjusted mortality for all patients and for patients without significant comorbidity or frailty, respectively. (C) and (D) show 17-y unadjusted mortality for all patients and for patients without significant comorbidity or frailty. The reference category is patients with a BMI of 20 kg/m^2^. In each panel, the black solid line denotes the estimated HR, and gray shading indicates the 95% confidence limits.
(TIF)

S2 Fig Adjusted 1-y and 17-y hazard ratios for underweight versus normal weight patients stratified by sex and age among patients without significant comorbidity or frailty. Adjusted 1-y (A) and 17-y (B) HRs. The reference category is patients with a BMI of 20 kg/m^2^. Analyses were adjusted for patient demographics (age, sex, race), cardiovascular risk factors (diabetes, hypertension, smoking, prior CAD), comorbidities (CHF, COPD, CVA/stroke, cirrhosis/liver disease, CKD, HIV or immunocompromised state, cancer, Alzheimer disease/dementia, terminal illness), markers of nutritional status (anemia, hypoalbuminemia), measures of frailty (admission from an SNF, mobility on admission, urinary continence on admission), clinical presentation (Killip classification, systolic blood pressure, heart rate, ST-elevation AMI, anterior infarction, cardiac arrest on admission, renal insufficiency), and treatment (PCI or CABG within the first 30 d of admission, fibrinolytic therapy, aspirin on admission, and beta-blockers on admission).
(TIF)

S1 Protocol Changes (DOCX)

## Abbreviations

AMI: acute myocardial infarction
BMI: body mass index
CABG: coronary artery bypass grafting
CAD: coronary artery disease
CHF: congestive heart failure
CKD: chronic kidney disease
COPD: chronic obstructive pulmonary disease
CVA: cerebrovascular accident
HIV: human immunodeficiency virus
HR: hazard ratio
PCI: percutaneous coronary intervention
SNF: skilled nursing facility
