# Correction: Underweight, Markers of Cachexia, and Mortality in Acute Myocardial Infarction: A Prospective Cohort Study of Elderly Medicare Beneficiaries

**DOI:** 10.1371/journal.pmed.1002061

**Published:** 2016-06-08

**Authors:** The PLOS Medicine Staff

## Abstract

This article was republished on May 19th, 2016 to correct poor figure quality in the PDF version, which was introduced during the typesetting process.

## Notice of Republication

This article was republished on May 19^th^, 2016 to correct poor figure quality in the PDF version, which was introduced during the typesetting process. The publisher apologizes for these issues with the figure quality. Please download this article again to view the updated version.
