# Supplementary material for: Underweight, Markers of Cachexia, and Mortality in Acute Myocardial Infarction: A Prospective Cohort Study of Elderly Medicare Beneficiaries
Source: PLoS Med. 2016 Apr 19;13(4):e1001998. doi: 10.1371/journal.pmed.1001998 (PMC4836735; doi:10.1371/journal.pmed.1001998)
Supplement: S1 STROBE checklist — (DOCX) [file pmed.1001998.s001.docx]

STROBE Statement—checklist of items that should be included in reports of observational studies

|  | | Item No. | Recommendation | | | Section, Paragraph No. | | | | | Relevant text from manuscript | | | | | | |  |  |
| --- | --- | --- | --- | --- | --- | --- | --- | --- | --- | --- | --- | --- | --- | --- | --- | --- | --- | --- | --- |
| **Title and abstract** | | 1 | (*a*) Indicate the study’s design with a commonly used term in the title or the abstract | | | Abstract, 2 | | | | | Abstract: “a cohort-based study of Medicare beneficiaries hospitalized for AMI” | | | | | | |  |  |
|  |  |  | (*b*) Provide in the abstract an informative and balanced summary of what was done and what was found | | | Abstract, 2-3 | | | | | **Methods and Findings:** We analyzed data from the Cooperative Cardiovascular Project, a cohort-based study of Medicare beneficiaries hospitalized for AMI between 1994-1996 with 17 years of follow-up and detailed clinical information to compare short and long-term mortality in underweight and normal weight patients (n=57,574). We used Cox proportional hazards regression to investigate the association of low BMI with 30-day, 1-year, 5-year, and 17-year mortality after AMI while adjusting for patient comorbidities, frailty measures, and laboratory markers associated with cachexia. We also repeated the analyses in a subset of patients without significant comorbidity or frailty.  Of the 57,574 patients with AMI included in this cohort, 5,678 (9.8%) were underweight and 51,896 (90.2%) were normal weight. Underweight patients were older, on average, than normal weight patients and had a higher prevalence of most comorbidities and measures of frailty. Crude mortality was significantly higher for underweight patients than normal weight patients at 30 days (25.2% vs. 16.4%, p<0.001), 1 year (51.3% vs. 33.8%, p<0.001), 5 years (79.2% vs. 59.4%, p<0.001), and 17 years (98.3% vs. 94.0%, p<0.001). After adjustment, underweight patients had 13% higher risk of 30-day death and 26% higher risk of death than normal weight patients over 17 years (30-day HR 1.13, 95% confidence interval (CI): 1.07-1.20; 17-year HR 1.26, 95% CI: 1.23-1.30). Survival curves for underweight and normal weight patients separated early and remained separate over 17-years, suggesting that underweight patients remained at a significant survival disadvantage over time. Similar findings were observed among the subset of patients without comorbidity. Underweight patients without comorbidity had similar 30-day adjusted mortality but 21% higher risk of death over the long-term than normal weight patients (30-day HR 1.08, 95% CI: 0.93-1.26; 17-year HR 1.21, 95% CI: 1.14-1.29). The adverse effects of low BMI were greatest in patients with very low BMIs. The major limitation of this study was the use of surrogate markers of frailty and comorbid conditions to identify patients at highest risk for cachexia rather than clear diagnostic criteria for cachexia. | | | | | | |  |  |
| Introduction | | | | | | | | | | |  | | | |  |  |  |  |  |
| Background/rationale | | 2 | Explain the scientific background and rationale for the investigation being reported | | | Introduction, 1 | | | | | Prior studies have largely attributed the excess mortality in underweight patients to confounding by cachexia, defined as unintentional weight loss, muscle atrophy, and fatigue that occur in the setting of chronic disease[3,9-12]; however, most studies lack information on measures of cachexia and thus are unable to test this hypothesis. As a result, it is unclear whether low body mass index (BMI) is a marker of generalized illness and risk or represents an independent risk factor worthy of attention in its own right. | | | | | | |  |  |
| Objectives | | 3 | State specific objectives, including any prespecified hypotheses | | | Introduction, 4 | | | | | Accordingly, we sought to further delineate the relationship between low BMI, cachexia, and mortality after AMI. We used detailed chart-abstracted data from a large cohort of Medicare beneficiaries with AMI to compare short and long-term mortality in underweight and normal weight patients while adjusting for numerous patient comorbidities, frailty measures, and laboratory markers associated with cachexia. In addition we repeated the analyses in a subset of patients without significant chronic illness. We posed two questions: 1) does comprehensive risk adjustment for comorbid illness and frailty measures explain the higher mortality after AMI in underweight patients, and 2) is the relationship between underweight and mortality also observed in patients with AMI who are otherwise without significant chronic illness and are presumably free of cachexia? Finally, we examined interactions between sex and age with underweight to determine whether the effect of underweight varies by other patient characteristics. | | | | | | |  |  |
| Methods | | | | | | | | | | |  | | | |  |  |  |  |  |
| Study design | | 4 | Present key elements of study design early in the paper | | | Methods, 1 | | | | | In brief, the CCP sampled fee-for-service Medicare beneficiaries hospitalized with a principal discharge diagnosis code of AMI (*ICD-9-CM code 410)* from acute-care nongovernmental hospitals in the U.S. between January 1994 and February 1996. Trained personnel performed the detailed medical record abstraction using an automated system to ensure standardization of techniques. Data quality was monitored by random reabstractions, assessment of reliability statistics, and focused abstractor training. | | | | | | |  |  |
| Setting | | 5 | Describe the setting, locations, and relevant dates, including periods of recruitment, exposure, follow-up, and data collection | | | Methods, 1, 4 | | | | | In brief, the CCP sampled fee-for-service Medicare beneficiaries hospitalized with a principal discharge diagnosis code of AMI (*ICD-9-CM code 410)* from acute-care nongovernmental hospitals in the U.S. between January 1994 and February 1996. Trained personnel performed the detailed medical record abstraction using an automated system to ensure standardization of techniques. Data quality was monitored by random reabstractions, assessment of reliability statistics, and focused abstractor training.  The primary outcomes were mortality at 30 days, 1 year, 5 years, and 17 years calculated from admission. Vital status was ascertained over 17 years through linkage to the 1994-2012 Medicare Denominator Files, which provides complete death information on all beneficiaries enrolled in Medicare. | | | | | | |  |  |
| Participants | | 6 | (*a*) *Cohort study*—Give the eligibility criteria, and the sources and methods of selection of participants. Describe methods of follow-up  *Case-control study*—Give the eligibility criteria, and the sources and methods of case ascertainment and control selection. Give the rationale for the choice of cases and controls  *Cross-sectional study*—Give the eligibility criteria, and the sources and methods of selection of participants | | | Methods, 2, 4 | | | | | For this study, we limited our analysis to patients ≥65 years old who were hospitalized with AMI that could be confirmed by medical record. The diagnosis of AMI was confirmed by elevated cardiac enzymes (e.g. elevation of creatine kinase-MB level >5% of total creatine kinase or elevation of lactate dehydrogenase enzyme (LDH) level with isoenzyme reversal (LDH_1_>LDH_2_)) or the presence of at least 2 of the following: chest pain, 2-fold elevation in total creatine kinase, or diagnostic changes on electrocardiogram (e.g. ST-segment elevation or new pathological Q-waves). If patients were admitted more than once for AMI during the study period, we included only the first admission. Finally, we excluded patients with missing height (n=24,014) or weight (n=13,180) data because we could not calculate BMI for these patients.  The primary outcomes were mortality at 30 days, 1 year, 5 years, and 17 years calculated from admission. Vital status was ascertained over 17 years through linkage to the 1994-2012 Medicare Denominator Files, which provides complete death information on all beneficiaries enrolled in Medicare. | | | | | | |  |  |
|  |  |  | (*b*) *Cohort study*—For matched studies, give matching criteria and number of exposed and unexposed  *Case-control study*—For matched studies, give matching criteria and the number of controls per case | | | N/A | | | | | N/A | | | | | | |  |  |
| Variables | | 7 | Clearly define all outcomes, exposures, predictors, potential confounders, and effect modifiers. Give diagnostic criteria, if applicable | | | Methods, 3-5 | | | | | BMI values were calculated from patients’ chart documented height and weight at the time AMI. We used criteria from the Centers for Disease Control and Prevention to classify patients as underweight (BMI<18.5kg/m^2^) or normal weight (18.5kg/m^2^≤BMI<25kg/m^2^).  The primary outcomes were mortality at 30 days, 1 year, 5 years, and 17 years calculated from admission. Vital status was ascertained over 17 years through linkage to the 1994-2012 Medicare Denominator Files, which provides complete death information on all beneficiaries enrolled in Medicare.  Cachexia-related variables were identified using prior literature, clinical judgment, and face validity for their association with underweight, cachexia, and frailty. Specifically, we included comorbidities that are known to cause cachexia (i.e. congestive heart failure (CHF), chronic obstructive pulmonary disease (COPD), cerebrovascular accident or stroke, chronic liver disease, chronic kidney disease (CKD), infection with human immunodeficiency virus (HIV), cancer, dementia, and other terminal illnesses). Comorbidities were ascertained through chart-documented medical history information, which was part of the patient’s medical record or collected during the index admission. In addition, we included two laboratory markers of nutritional status extracted from patient charts: anemia (hematocrit <30%) and hypoalbuminemia (serum albumin <3g/dL) and three variables reflecting frailty prior to admission (admission from a skilled nursing facility (SNF), mobility, and urinary continence on admission). Mobility (walks independently, walks with assistance, unable to walk) and incontinence (continent, totally/occasionally incontinent, anuric) on admission were determined from provider notes and chart-documented impairments. We selected these variables because validated frailty scales have typically included some combination of activities of daily living or self-sufficiency[25-27], urinary continence[26-28], mobility[25,28-30], stamina[25,28], and cognitive functioning[27-30] to evaluate frailty. Although we lacked information on cognitive functioning and stamina, we incorporated assessments of mobility and continence, and we used residence at a skilled nursing facility as a proxy for self-sufficiency. Patients with missing systolic blood pressure were assigned the median value in the overall cohort and a dummy variable to denote missing. Patients with missing categorical variables (mobility, urinary continence, and PCI/CABG) were included in the model using dummy variables for missing data. | | | | | | |  |  |
| Data sources/ measurement | | 8* | For each variable of interest, give sources of data and details of methods of assessment (measurement). Describe comparability of assessment methods if there is more than one group | | | Methods, 1,3,4,5,5 | | | | | Trained personnel performed the detailed medical record abstraction using an automated system to ensure standardization of techniques. Data quality was monitored by random reabstractions, assessment of reliability statistics, and focused abstractor training.  BMI values were calculated from patients’ chart documented height and weight at the time AMI.  The primary outcomes were mortality at 30 days, 1 year, 5 years, and 17 years calculated from admission. Vital status was ascertained over 17 years through linkage to the 1994-2012 Medicare Denominator Files, which provides complete death information on all beneficiaries enrolled in Medicare.  Comorbidities were ascertained through chart-documented medical history information, which was part of the patient’s medical record or collected during the index admission.  Mobility (walks independently, walks with assistance, unable to walk) and incontinence (continent, totally/occasionally incontinent, anuric) on admission were determined from provider notes and chart-documented impairments. | | | | | | |  |  |
| Bias | | 9 | Describe any efforts to address potential sources of bias | | | N/A | | | | | N/A | | | | | | |  |  |
| Study size | | 10 | Explain how the study size was arrived at | | | Methods, 2 | | | | | For this study, we limited our analysis to patients ≥65 years old who were hospitalized with AMI that could be confirmed by medical record. The diagnosis of AMI was confirmed by elevated cardiac enzymes (e.g. elevation of creatine kinase-MB level >5% of total creatine kinase or elevation of lactate dehydrogenase enzyme (LDH) level with isoenzyme reversal (LDH_1_>LDH_2_)) or the presence of at least 2 of the following: chest pain, 2-fold elevation in total creatine kinase, or diagnostic changes on electrocardiogram (e.g. ST-segment elevation or new pathological Q-waves). If patients were admitted more than once for AMI during the study period, we included only the first admission. Finally, we excluded patients with missing height (n=24,014) or weight (n=13,180) data because we could not calculate BMI for these patients. | | | | | | |  |  |
| Quantitative variables | 11 | | | Explain how quantitative variables were handled in the analyses. If applicable, describe which groupings were chosen and why | | | | | Methods, 3 | | BMI values were calculated from patients’ chart documented height and weight at the time AMI. We used criteria from the Centers for Disease Control and Prevention to classify patients as underweight (BMI<18.5kg/m^2^) or normal weight (18.5kg/m^2^≤BMI<25kg/m^2^). | | | | | | |  |  |
| Statistical methods | 12 | | | (*a*) Describe all statistical methods, including those used to control for confounding | | | Methods, 6,7 | | | Baseline characteristics were compared between underweight and normal weight patients using chi-squared tests for categorical variables and student’s t-tests for continuous variables. To evaluate the relationship of underweight to short and long-term mortality, we performed two sets of analyses modeling BMI first as a categorical and then as a continuous variable. In analyses of BMI as a categorical variables, we used chi-squared tests, Kaplan-Meier curves with log-rank tests, and Cox proportional hazards regression to compare unadjusted and adjusted mortality at 30 days, 1, 5, and 17 years after AMI between normal weight and underweight patients. In addition, we calculated conditional hazard ratios for the intervals 0 to 30 days, 30 days to 1 year, 1 to 5 years, and 5 to 17 years to determine whether underweight patients were at higher risk of death early after AMI or accrued a survival disadvantage over time. Interaction terms between sex and age with underweight were tested in all models.  In the second set of analyses, we modeled BMI as a continuous variable to better characterize the shape of the association between low BMI with 1 and 17-year mortality. Specifically, we modeled the hazards of death relative to patients with BMI of 20kg/m^2^ using proportional hazards regression restricted cubic spline models with knots located at each BMI integer value[31,32]. This approach combines linear and nonlinear transformations of BMI at different sections of the BMI curve to identify the best-fitting transformations for the association between BMI and mortality. Models were then repeated adjusting for the same covariates above. | | | | | | |  |  |  |
|  |  |  |  | (*b*) Describe any methods used to examine subgroups and interactions | | | Methods, 8 | | | Finally, because multivariate adjustment may be insufficient to remove confounding by cachexia, we repeated the above analyses in a subset of patients without significant comorbidity or frailty (n=20,587). Specifically, we excluded patients with CHF, COPD, stroke, liver disease, CKD, HIV, cancer, dementia, terminal illness, anemia, and hypoalbuminemia, patients admitted from SNFs, and patients with mobility issues or incontinence. | | | | | | |  |  |  |
|  |  |  |  | (*c*) Explain how missing data were addressed | | | Methods, 5 | | | Patients with missing systolic blood pressure were assigned the median value in the overall cohort and a dummy variable to denote missing. Patients with missing categorical variables (mobility, urinary continence, and PCI/CABG) were included in the model using dummy variables for missing data. | | | | | | |  |  |  |
|  |  |  |  | (*d*) *Cohort study*—If applicable, explain how loss to follow-up was addressed  *Case-control study*—If applicable, explain how matching of cases and controls was addressed  *Cross-sectional study*—If applicable, describe analytical methods taking account of sampling strategy | | | Methods, 4 | | | Vital status was ascertained over 17 years through linkage to the 1994-2012 Medicare Denominator Files, which provides complete death information on all beneficiaries enrolled in Medicare. | | | | | | |  |  |  |
|  |  |  |  | € Describe any sensitivity analyses | | | N/A | | |  | | | | | | |  |  |  |
| Results | | | | | | | | | | | | | | | | | |  |  |
| Participants | 13* | | | (a) Report numbers of individuals at each stage of study—eg numbers potentially eligible, examined for eligibility, confirmed eligible, included in the study, completing follow-up, and analysed | | | Methods, 2 | | | | | For this study, we limited our analysis to patients ≥65 years old who were hospitalized with AMI that could be confirmed by medical record. The diagnosis of AMI was confirmed by elevated cardiac enzymes (e.g. elevation of creatine kinase-MB level >5% of total creatine kinase or elevation of lactate dehydrogenase enzyme (LDH) level with isoenzyme reversal (LDH_1_>LDH_2_)) or the presence of at least 2 of the following: chest pain, 2-fold elevation in total creatine kinase, or diagnostic changes on electrocardiogram (e.g. ST-segment elevation or new pathological Q-waves). If patients were admitted more than once for AMI during the study period, we included only the first admission. Finally, we excluded patients with missing height (n=24,014) or weight (n=13,180) data because we could not calculate BMI for these patients. | | | |  |  |  |  |
|  |  |  |  | (b) Give reasons for non-participation at each stage | | | N/A | | | | |  | | | |  |  |  |  |
|  |  |  |  | © Consider use of a flow diagram | | | N/A | | | | |  | | | |  |  |  |  |
| Descriptive data | 14* | | | (a) Give characteristics of study participants (eg demographic, clinical, social) and information on exposures and potential confounders | | | Results, 1,2  Table 1 | | | | | Our sample included 5,678 (9.8%) underweight patients and 51,896 (90.2%) normal weight patients. Underweight and normal weight patients represented 44% of all eligible patients. Compared with patients with recorded BMI values, patients with missing BMI were on average older (mean age 78.6 vs. 76.0, p<0.001). In addition, they were more likely to be admitted from nursing homes (14.0% vs. 5.0%, p<0.001) and less likely to be mobile (68.1% vs. 80.7%, p<0.001) or continent (82.0% vs. 92.0%, p<0.001) on admission. Patients with missing BMI had higher in-hospital and 17-year mortality rates (in-hospital: 27.0% vs. 11.9%, p<0.001; 17-year: 96.1% vs. 92.3%, p<0.001).  Underweight patients were older, on average, and had a greater percentage of women than normal weight patients (**Table 1**). Although underweight patients had a lower prevalence of diabetes, hypertension, and prior coronary artery disease, they had significantly higher rates of smoking and nearly all other comorbidities, including CHF, COPD, stroke, CKD, cancer, dementia, anemia, and poor nutritional status. They were also more likely to be admitted from SNFs and to have decreased mobility and urinary continence on admission (**Table 1**). Underweight patients were significantly less likely to receive guideline-based therapies on admission including aspirin, beta-blockers, primary reperfusion, and revascularization procedures. | | | |  |  |  |  |
|  |  |  |  | (b) Indicate number of participants with missing data for each variable of interest | | | Table 1 | | | | | Table 1 | | | |  |  |  |  |
|  |  |  |  | (c) *Cohort study*—Summarise follow-up time (eg, average and total amount) | | | Methods, 4 | | | | | Vital status was ascertained over 17 years through linkage to the 1994-2012 Medicare Denominator Files, which provides complete death information on all beneficiaries enrolled in Medicare. | | | |  |  |  |  |
| Outcome data | 15* | | | *Cohort study*—Report numbers of outcome events or summary measures over time | | | Results, 3  Table 2 | | | | | In-hospital mortality was significantly higher for underweight patients compared with normal weight patients; however, rates of most other in-hospital complications were similar (**Table 1**). Crude mortality was significantly higher for underweight patients than normal weight patients at 30 days (25.2% vs. 16.4%), 1 year (51.3% vs. 33.8%), 5 years (79.2% vs. 59.4%), and 17 years (98.3% vs. 94.0%) (all p<0.001) (**Fig 1 and Table 1**) | | | |  |  |  |  |
|  |  |  |  | *Case-control study—*Report numbers in each exposure category, or summary measures of exposure | | | N/A | | | | | N/A | | | |  |  |  |  |
|  |  |  |  | *Cross-sectional study—*Report numbers of outcome events or summary measures | | | N/A | | | | | N/A | | | |  |  |  |  |
| Main results | 16 | | | (*a*) Give unadjusted estimates and, if applicable, confounder-adjusted estimates and their precision (eg, 95% confidence interval). Make clear which confounders were adjusted for and why they were included | | | Results, 3,4,6  Table 2,  Figures 1,2,3  S1_Fig | | | | | Conditional hazard ratios showed divergence of the survival curves over all 17 years of follow-up, suggesting that underweight patients remained at a significant survival disadvantage over time (**Table 2**). After adjustment for patient and treatment characteristics, underweight patients remained at a significant survival disadvantage across all follow-up time points and the curves diverged early and remained separate over 17 years. Underweight patients had a 13% greater risk of death within the first 30 days and a 26% greater risk of death over the full 17 years of follow-up (30-day adjusted HR: 1.13, 95%CI: 1.07-1.20; 17-year adjusted HR: 1.26, 95%CI: 1.23-1.30) (**Table 2**).  When BMI was examined as a continuous variable, there was an inverse relationship between BMI and the hazards of death at both 1 and 17 years. The highest risk of death was observed in those with very low BMI (<17kg/m^2^) and the lowest risk in those with BMIs in the upper range of normal (>24kg/m^2^). This relationship persisted after adjustment **(Fig 2, and S1 Fig)**.  Underweight was associated with increased risk of death in both sexes and at all ages at both 1 and 17 years; however, the relationship between underweight and mortality was stronger in men and younger patients (65-75 years of age) (p-values for interactions <0.01) (**Fig 3**). | | | |  |  |  |  |
|  |  |  |  | (*b*) Report category boundaries when continuous variables were categorized | | | N/A | | | | | N/A | | | |  |  |  |  |
|  |  |  |  | (*c*) If relevant, consider translating estimates of relative risk into absolute risk for a meaningful time period | | | N/A | | | | | N/A | | | |  |  |  |  |
| Other analyses | 17 | | | Report other analyses done—eg analyses of subgroups and interactions, and sensitivity analyses | | | Results, 5,6  Table 2,  Figures 1,2  S2_Fig | | | | | | | To further reduce the potential for confounding by cachexia, we repeated the analyses in a subset of underweight and normal weight patients without significant comorbidities or markers of frailty. Compared with the previous analyses, a smaller percentage of the cohort was classified as underweight (n=1081, 5.2%). However, baseline comparisons between underweight and normal weight patients in this subset were similar to the previous analyses (**Table 1**). Crude mortality rates were higher for underweight patients across all follow-up time points (30-day: 16.7% vs. 10.7%; 1-year: 29.9% vs. 19.0%; 5-year: 53.8% vs. 37.3%; 17-year: 94.5% vs. 87.7%), (**Fig 1 and Table 1**) and risk estimates were similar to those in analyses of all patients (**Table 2**). Conditional hazard ratios again showed early divergence of the survival curves, which remained separate over all 17 years of follow-up (**Table 2**). After adjustment, underweight and normal weight patients had a similar risk of 30-day mortality (HR 1.08, 95% confidence interval (CI): 0.93-1.26); however, the long-term risk of death in underweight patients remained significantly higher than that in normal weight patients (17-year HR 1.21, 95% CI: 1.14-1.29). Similarly, when BMI was modeled as a continuous variable, we observed an inverse relationship between BMI and the hazards of death; however, the magnitudes of the hazard ratios for low BMIs were smaller in the subset of patients without significant comorbidity than in all patients (**Fig 2**).  After limiting the cohort to patients without significant comorbidity, however, only the interaction between underweight and age on 17-year mortality as significant (**S2 Fig**). | | |  |  |  |
| Discussion | | | | | | | | | | | | | | | | | | | |
| Key results | 18 | | | | Summarise key results with reference to study objectives | | | Discussion, 1 | | | | | Using detailed clinical data from the largest study of elderly patients with AMI, we found that low BMI was associated with increased short and long-term mortality after AMI. Underweight patients had a 61 to 73% higher crude risk of death compared with normal weight patients at all follow-up time points. The survival curves for underweight and normal weight patients diverged early and remained separate over all 17 years of follow-up, suggesting that underweight patients accrued a survival disadvantage over time. Although adjustment for markers of cachexia (comorbid conditions, measures of frailty, and nutritional status) as well as other patient and treatment characteristics attenuated some of the excess risk in underweight patients, underweight patients still had a 13 to 27% higher risk of death compared with normal weight patients. Furthermore, when we restricted the cohort to a subset of patients without significant comorbidity or frailty, underweight patients continued to have an 8 to 22% higher risk of death than normal weight patients. | | | | | |  |
| Limitations | 19 | | | | Discuss limitations of the study, taking into account sources of potential bias or imprecision. Discuss both direction and magnitude of any potential bias | | Discussion, 9 | | | | | | | Our study has some limitations. First, we were unable to determine which patients met criteria for cachexia. Although many criteria exist, most include current BMI or recent weight loss, symptoms of fatigue or anorexia, and biochemical markers in the setting of chronic disease. Like many other studies, we lacked information on recent weight trends, and thus relied on other markers of frailty, nutritional status, and comorbid conditions to identify patients at highest risk for cachexia. Second, many factors other than cachexia may contribute to low BMI in elderly patients including malnutrition, sarcopenia, genetics, or increased metabolic demands. We lacked information on nutritional status and recent weight loss and therefore were unable to determine the primary cause of low BMI in underweight patients. Thus, it is possible that the effect of underweight on post-AMI mortality varies by cause. Future studies should evaluate the effect of nutritional status and lifetime changes in BMI on the relationship between underweight and mortality after AMI. Third, we used patient BMI measured at the index hospitalization. Reports from other AMI cohorts have been mixed with some reporting minimal weight changes in the year after AMI[45] and others reporting sizeable weight gains or losses[46,47], although these studies have largely been performed in cohorts of heavier patients. Fourth, we excluded 27,690 patients (17.5% of the initial sample) for missing BMI data. Because patients with missing BMI data had higher short and long-term mortality rate than patients in our sample, our cohort may be healthier than the general AMI population. Fifth, we lacked information on cause of death and thus could not identify the cause of the excess deaths. Finally, we used dummy variables for missing data rather imputing missing values. Although this approach is not preferred because patients with missing data can have dissimilar values[48], we chose this approach due to the high computational cost of multiple imputations and the low missing data rates. | | | | | |
| Interpretation | 20 | | | | Give a cautious overall interpretation of results considering objectives, limitations, multiplicity of analyses, results from similar studies, and other relevant evidence | | Discussion, 7 | | | | | | | Clinically, our findings imply that underweight patients may benefit from treatment strategies that focus on promoting nutritional status and weight gain, regardless of the reason for their low BMI. Such strategies may include inpatient caloric supplementation and outpatient nutritional consults in addition to pharmacotherapy. Recently, pharmaceutical agents, such as Megestrol acetate, medroxyprogesterone, ghrelin, and omega-3-fatty acid have been used to promote weight gain and improve survival in the setting of cancer and cardiac cachexia[42-44]. Such agents may benefit underweight patients with and without cachexia after AMI; however, trials are needed to test whether use of these therapies improves weight gain in patients with AMI and whether weight gain in underweight patients improves survival after AMI. Similarly, a better understanding of why underweight patients are at increased risk of mortality after AMI, including the physiologic, therapeutic, and systems level causes, would help us to better target therapies to improve outcomes in these patients. | | | | | |
| Generalisability | 21 | | | | Discuss the generalisability (external validity) of the study results | | Discussion, 6 | | | | | | | To our knowledge, this is the first study to report differences in the effect of underweight on mortality after AMI by age and sex. Although the mechanisms underlying these differences are unclear, it is possible that lower BMI in men reflects a more malnourished or cachetic state since men typically have higher BMIs and lean body mass than women. We also found that underweight was more potent in younger patients. Although these differences by age may reflect our ability to detect larger differences in mortality in younger patients with higher survival, it is also possible that older age acts as an equalizer of risk because older underweight and normal weight patients have reduced physiologic reserve to overcome acute events like AMI[39-41]. | | | | | |
| Other information | |  | | | | | | | | | | | | | | | | | |
| Funding | 22 | | | | Give the source of funding and the role of the funders for the present study and, if applicable, for the original study on which the present article is based | | N/A | | | | | | | EMB is supported by an F30 Training grant F30HL120498-01A1 from the National Heart, Lung, and Blood Institute. HMK is supported by grant U01 HL105270-05 (Center for Cardiovascular Outcomes Research at Yale University) from the National Heart, Lung, and Blood Institute. HAK reports no financial disclosures. The funders had no role in study design, data collection and analysis, decision to publish, or preparation of the manuscript. | | | | | |

*Give information separately for cases and controls in case-control studies and, if applicable, for exposed and unexposed groups in cohort and cross-sectional studies.

**Note:** An Explanation and Elaboration article discusses each checklist item and gives methodological background and published examples of transparent reporting. The STROBE checklist is best used in conjunction with this article (freely available on the Web sites of PLoS Medicine at http://www.plosmedicine.org/, Annals of Internal Medicine at http://www.annals.org/, and Epidemiology at http://www.epidem.com/). Information on the STROBE Initiative is available at www.strobe-statement.org.
