# Supplementary figures and images for: Underweight, Markers of Cachexia, and Mortality in Acute Myocardial Infarction: A Prospective Cohort Study of Elderly Medicare Beneficiaries
Source: PLoS Med. 2016 Apr 19;13(4):e1001998. doi: 10.1371/journal.pmed.1001998 (PMC4836735; doi:10.1371/journal.pmed.1001998)

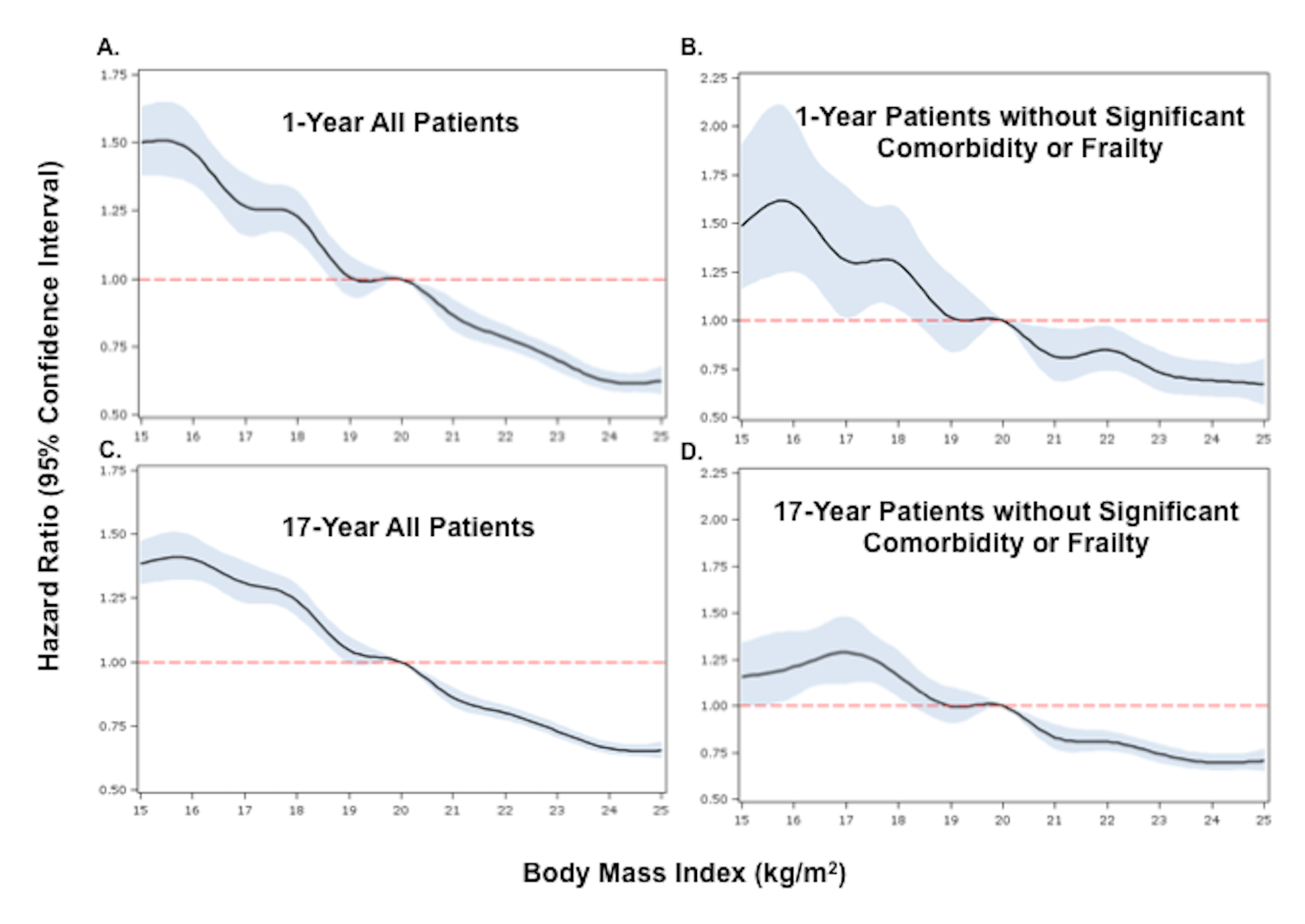

Supplement: S1 Fig — (A) and (B) show 1-y unadjusted mortality for all patients and for patients without significant comorbidity or frailty, respectively. (C) and (D) show 17-y unadjusted mortality for all patients and for patients without significant comorbidity or frailty. The reference category is patients with a BMI of 20 kg/m2. In each panel, the black solid line denotes the estimated HR, and gray shading indicates the 95% confidence limits. (TIF) [file pmed.1001998.s002.tif]

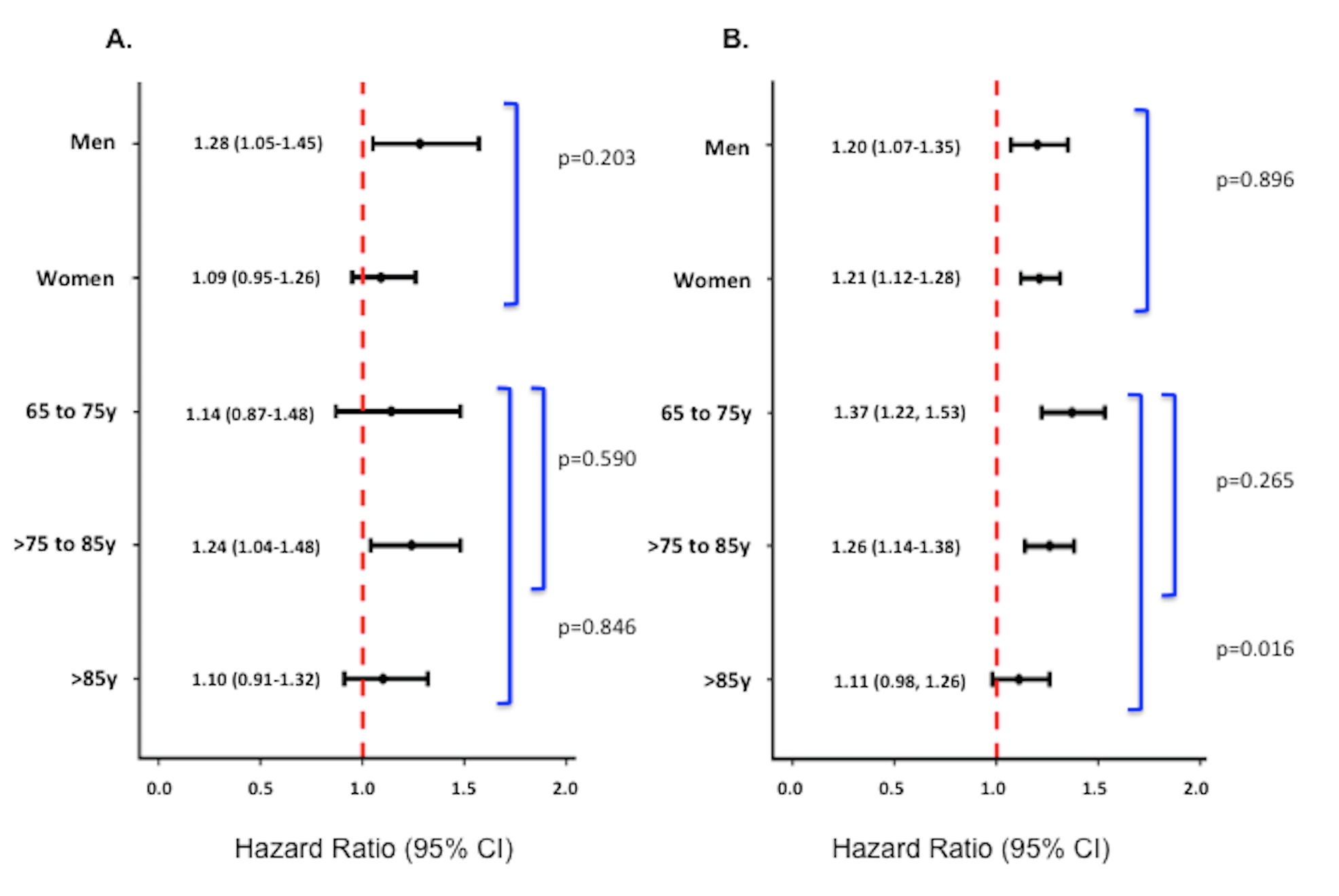

Supplement: S2 Fig — Adjusted 1-y (A) and 17-y (B) HRs. The reference category is patients with a BMI of 20 kg/m2. Analyses were adjusted for patient demographics (age, sex, race), cardiovascular risk factors (diabetes, hypertension, smoking, prior CAD), comorbidities (CHF, COPD, CVA/stroke, cirrhosis/liver disease, CKD, HIV or immunocompromised state, cancer, Alzheimer disease/dementia, terminal illness), markers of nutritional status (anemia, hypoalbuminemia), measures of frailty (admission from an SNF, mobility on admission, urinary continence on admission), clinical presentation (Killip classification, systolic blood pressure, heart rate, ST-elevation AMI, anterior infarction, cardiac arrest on admission, renal insufficiency), and treatment (PCI or CABG within the first 30 d of admission, fibrinolytic therapy, aspirin on admission, and beta-blockers on admission). (TIF) [file pmed.1001998.s003.tif]
