# Supplementary material for: Underweight, Markers of Cachexia, and Mortality in Acute Myocardial Infarction: A Prospective Cohort Study of Elderly Medicare Beneficiaries
Source: PLoS Med. 2016 Apr 19;13(4):e1001998. doi: 10.1371/journal.pmed.1001998 (PMC4836735; doi:10.1371/journal.pmed.1001998)
Supplement: S1 Protocol Changes — (DOCX) [file pmed.1001998.s004.docx]

**Changes to Proposal:** Relationship between Excess and Underweight with Life Expectancy after Acute Myocardial Infarction (AMI)

**Authors:**

Emily Bucholz

Hannah Krumholz

Harlan Krumholz

We initially followed the proposal, performing all crude life expectancy and years of potential life lost calculations for over and underweight patients. These are shown below in figures 1 and 2. However, we realized that underweight patients were a particularly high-risk group with significantly higher mortality and shorter life expectancy than other BMI groups. Given these findings, we felt that it would be too much to present both underweight and overweight patients in the same paper and that the underweight analyses warranted a more in depth discussion of potential mechanisms. We therefore decided to split the study into two parts: one examining the effects of overweight and obesity and the other examining the effects of underweight after AMI.

Similarly, when deciding how to best present the underweight analyses, we felt that modeling BMI as both categorical and continuous variables were warranted in order to determine how the risk of mortality changed at lower BMIs. In addition, we saw that certain subgroups of patients (women and older patients) were at particularly high risk of underweight. To better understand whether the effect of underweight differed across these patient subgroups, we evaluated interactions with age and gender. Throughout this process, we kept the same covariates identified a priori and only evaluated age and sex as potential effect modifiers. All analytic decisions were made a priori before running analyses.

A brief timeline of deviations from the protocol is given below:

October 2014: Proposal drafted

January 2015: Preliminary analyses completed including Cox models, life expectancy estimates, and years of potential life lost estimates

February 2015: Study split into obesity and underweight analyses to allow further exploration of why underweight patients were at particularly high risk after AMI

April 2015: Life expectancy estimates removed from underweight analyses

April 2015: Spline curves added to underweight study

April 2015: Age and sex-specific subgroup analyses were performed

May 2015: All figures/tables were assembled and manuscript drafting started

**Working Title: Relationship between Excess and Underweight with Life Expectancy after Acute Myocardial Infarction (AMI)**

**Authors:**

Emily Bucholz

Hannah Krumholz

Harlan Krumholz

**INTRODUCTION**

An “obesity paradox” has been described in patients with acute myocardial infarction (AMI), whereby obese and overweight patients have a lower risk of short-term mortality after AMI than normal weight patients. Whether obese patients have improved long-term survival after AMI is not known, however. Although studies of short-term mortality have consistently reported lower risk of mortality for obese patients,[[1-5](#_ENREF_1)] the few studies examining long-term mortality after AMI have reported mixed results. Some studies have found lower long-term mortality for obese patients,[[6-9](#_ENREF_6)] whereas others have reported no differences across obesity levels.[[10-12](#_ENREF_10)] The discrepancies in study results are likely explained by differences in length of follow-up and covariate adjustment. In general, studies reporting an obesity paradox have had shorter follow-up periods (2-3 years)[[6-9](#_ENREF_6)] than studies finding no effect (8 years)[[11](#_ENREF_11), [12](#_ENREF_12)], suggesting that the relationship between BMI and mortality may attenuate over time. In addition, prior studies have suggested that the obesity paradox may be due to the deleterious effects of cachexia in normal weight patients, rather than the beneficial effects of obesity.[[1](#_ENREF_1), [13](#_ENREF_13), [14](#_ENREF_14)]

Similarly, underweight patients have significantly higher mortality after AMI than normal weight patients. Prior studies have largely attributed the excess mortality in underweight patients to confounding by cachexia rather than the direct effects of low BMI; however, most studies lack information on measures of cachexia or comorbid illness and thus are unable to test this hypothesis. As a result, it is unclear whether BMI is independently predictive of excess mortality after AMI or is simply a marker of cachexia, severe illness, or frailty.

Measuring the years of life gained or lost due to low and high BMI is one way to quantify the absolute benefit or burden of underweight and obesity in patients with AMI. This value compares the number of years of life lived after AMI by underweight or obese patients to that of normal weight patients and evaluates the effects of underweight and obesity over the entire remaining lifespan. In addition, it allows us to evaluate the impact of underweight and obesity across all ages and multiple patient subgroups to determine whether the burden of underweight or obesity varies by patient characteristics. This information is essential for clinicians to educate underweight and obese patients on the harms and benefits of low and excess weight after AMI and to appropriately advise these patients on strategies for mitigating their long-term risk.

Answering these questions requires the examination of BMI with both short and long-term mortality while controlling for factors that may be associated with both BMI and cachexia. This in turn requires a large comprehensive database of AMI patients that contains detailed clinical information and long-term follow-up to measure the years of life lost or gained after AMI due to high or low BMI. With over 17 years of complete follow-up and rich clinical information on a nationally representative sample of over 230,000 AMI patients, the Cooperative Cardiovascular Project (CCP) provides this opportunity. In accordance, we undertook the following study to estimate the years of life lost or gained due to high or low BMI in patients with AMI and thus characterize the long-term benefits or harms of obesity and underweight after AMI.

**SPECIFIC AIMS**

1. To characterize differences in markers of cachexia, illness, and frailty at the time of AMI across BMI groups.
2. To evaluate the relationship between BMI and survival after AMI using three approaches:
   1. Cox proportional hazards curves
   2. Life expectancy estimation
   3. Years of potential life lost after AMI from excess and underweight
3. To evaluate the independent relationship between BMI and survival after AMI while controlling for comorbid illness and additional markers of cachexia and frailty.
4. To determine whether the relationship between BMI and survival after AMI differs by age.

**METHODS: Analytic Approach**

***Study Sample***

Dataset: Cooperative Cardiovascular Project

Inclusion Criteria:

1. Patients aged ≥65 years
2. AMI defined by discharge diagnosis of an AMI and:
   1. Either a creatine kinase MB level >0.05 or a lactate dehydrodgenase level >1.5x normal and LDH-1 level higher than LDH-2 level
   2. OR, 2 of the following 3 criteria: chest pain, 2-fold elevation of the CK level, or evidence of AMI on EKG
3. Only first confirmed AMI hospitalizations in the CCP to avoid counting patients twice
4. Patients must have both height and weight recorded at baseline to calculate BMI

***Variable Definitions***

Body Mass Index: Calculated as ratio of weight (kg) to height-squared (m^2^). Categories defined using Centers for Disease Control and Prevention (CDC) criteria.

1. Underweight: <18.5kg/m^2^
2. Normal weight: 18-24.9kg/m^2^
3. Overweight: 25-29.9kg/m^2^
4. Obese: 30-34.5kg/m^2^
5. Morbidly obese: ≥35kg/m^2^

***Outcome Measurement: Mortality***

For data on mortality, the CCP will be linked to the Social Security Death Master File (SS-DMF), which contains information on deceased persons including social security number (SSN) and date of death. Data will be linked using HIC numbers and deidentified thereafter. Length of survival will be calculated from the date of admission.

**Outcome Definitions**

1. Life Expectancy: Mean survival after AMI or the area under the survival curve.
2. Years of Potential Life Lost: Mean difference in life expectancy between underweight, overweight, obese, and morbidly obese patients from that of normal weight patients.

**Covariate Definitions and Units**

1. Demographics
   1. Age (years)
   2. Gender (female vs male)
   3. Race (nonwhite vs white)
   4. Median household income ($): ZIP-code level median household income percentile using 1990 census data
2. Risk factors
   1. Diabetes mellitus
   2. Hypertension
   3. Smoker
   4. Prior coronary events (including AMI, percutaneous coronary intervention, or coronary artery bypass grafting)
   5. Peripheral vascular disease
3. Comorbidities
   1. Congestive heart failure
   2. Chronic obstructive pulmonary disease
   3. Cerebrovascular accident/stroke
   4. Chronic kidney disease
   5. HIV/immunocompromised
   6. Cancer
   7. Alzheimer’s demention
   8. Anemia: hematocrit <30% on admission labs
   9. Hypoalbuminemia: albumin <3g/dL on admission labs
4. Pre-Admission Status
   1. Admitted from nursing home
   2. Mobility at admission (walks independently vs walks with assistance vs unable to walk)
   3. Urinary continence at admission (continent vs occasionally incontinent vs totally incontinent vs anuric)
5. Clinical Presentation
   1. Killip Class (I vs. II vs. III vs. IV)
   2. Systolic blood pressure on admission (mmHg)
   3. Heart rate on admission (bpm)
   4. ST-elevation AMI: determined using initial ECG
   5. Anterior infarction: determined using initial ECG
   6. Cardiac arrest on admission
   7. Renal insufficiency: serum creatinine >1.5mg/dL
6. Treatment
   1. Receipt of PCI/CABG within first 30 days from admission
   2. Receipt of fibrinolytic therapy during admission
   3. Receipt of aspirin within 48 hours of admission among patients without contraindications
   4. Receipt of beta-blockers within 48 hours of admission among patients without contraindications

***Statistical Analyses***

We will first compare baseline characteristics between BMI categories using X^2^ tests for categorical variables and student’s t-tests for continuous variables. We will then calculate unadjusted age-specific life expectancy using Cox proportional hazards regression with extrapolation using exponential models. These models have been used in previous work. The steps are outlined below.

1. First, we will fit a Cox proportional hazards model that includes covariates age, BMI category, and their pairwise interactions. We will use a semiparametric approach because it provides the best fit to the data using AIC and likelihood ratio statistics compared with parametric models such as the Weibull or Gompertz. Proportional hazards assumptions will be checked using Schoenfeld residuals, examined graphically and tested formally.
2. Second, we will plot the 17-year expected survival curves from the Cox models for each age and BMI combination and then extrapolate the curves to age 100 using exponential models. Age 100 has been chosen because the Centers for Disease Control and Prevention uses thi sage as the upper limit in its life-table analyses for general population life expectancy. We have chosen an exponential model because we do not have information on the shape of the hazard function beyond 17 years and exponential models offer a conservative approach. The constant hazard for the exponential model will be the average hazard over the last 2 years of available follow-up.
3. Third, mean life expectancy estimates will be calculated by adding the areas under the Cox and exponential survival functions. Ninety-five percent confidence intervals for the mean will be calculated using the same process for the upper and lower confidence bounds of the expected survival curves.

YPLLs will be calculated by subtracting mean age-specific life expectancy estimates in overweight, obese, morbidly obese, and underweight patients from those of normal weight patients of the same age to determine the years of life lost after AMI attributable to excess and underweight.

To determine whether differences in demographics, cardiovascular risk factors, comorbidities, pre-admission status, clinical presentation, and treatment might explain the observed differences in survival across BMI categories, we will repeat the life expectancy and YPLL calculation while adjusting for those covariates given above. We will use age-specific frequencies or mean values to plot the BMI- and age-specific expected survival curves from the models. This approach forces same-aged patients in different BMI classes to have similar values of baseline characteristics and thus allows us to compare life expectancy across BMI categories at fixed covariate values. Table shells and mock figures are given below.

**PROPOSED TABLES**

**Table 1.** Sample characteristics by body mass index category (n=130,659)

| **Characteristics** | **Underweight**  **N=5678** | **Normal Weight**  **N=51,896** | **Overweight**  **N=48,422** | **Obese**  **N=17,822** | **Morbidly Obese**  **N=6841** | **p-value** |
| --- | --- | --- | --- | --- | --- | --- |
| **Demographics** |  |  |  |  |  |  |
| Age, mean (SD) |  |  |  |  |  |  |
| Female |  |  |  |  |  |  |
| Nonwhite race |  |  |  |  |  |  |
| Median household income, mean (SD)  Missing |  |  |  |  |  |  |
| **Risk Factors** |  |  |  |  |  |  |
| Diabetes mellitus |  |  |  |  |  |  |
| Hypertension |  |  |  |  |  |  |
| Smoker |  |  |  |  |  |  |
| Prior coronary events (AMI, PCI, CABG) |  |  |  |  |  |  |
| Peripheral vascular disease |  |  |  |  |  |  |
| **Comorbidities** |  |  |  |  |  |  |
| Congestive heart failure |  |  |  |  |  |  |
| Chronic obstructive pulmonary disease |  |  |  |  |  |  |
| Cerebrovascular accident/stroke |  |  |  |  |  |  |
| Chronic kidney disease |  |  |  |  |  |  |
| HIV/Immunocompromised |  |  |  |  |  |  |
| Cancer |  |  |  |  |  |  |
| Alzheimer’s/Dementia |  |  |  |  |  |  |
| Anemia (Hct<30%) |  |  |  |  |  |  |
| Hypoalbuminemia (<3) |  |  |  |  |  |  |
| **Pre-Admission Status** |  |  |  |  |  |  |
| Admitted from nursing home |  |  |  |  |  |  |
| Mobility at admission  Walks independently  Walk with assistance  Unable to walk  Missing |  |  |  |  |  |  |
| Urinary continence at admission  Continent  Totally/Occasionally incontinent  Anuric  Missing |  |  |  |  |  |  |
| **Clinical Presentation** |  |  |  |  |  |  |
| Killip class >2 |  |  |  |  |  |  |
| Systolic blood pressure (mmHg), mean(SD)  Missing |  |  |  |  |  |  |
| Heart rate (bpm), mean (SD) |  |  |  |  |  |  |
| STEMI |  |  |  |  |  |  |
| Anterior infarction |  |  |  |  |  |  |
| Cardiac arrest on admission |  |  |  |  |  |  |
| Renal insufficiency |  |  |  |  |  |  |
| **Treatment** |  |  |  |  |  |  |
| PCI/CABG in first 30 days  Missing |  |  |  |  |  |  |
| Fibrinolytic therapy |  |  |  |  |  |  |
| Aspirin on admission of eligible patients |  |  |  |  |  |  |
| B-blockers on admission of eligible patients |  |  |  |  |  |  |
| **Crude Mortality Rates** |  |  |  |  |  |  |
| Hospital  30-day |  |  |  |  |  |  |
| 1-year |  |  |  |  |  |  |
| 5-year |  |  |  |  |  |  |
| 17-year |  |  |  |  |  |  |

Abbreviations: AMI, acute myocardial infarction; CABG, coronary artery bypass grafting; HIV, Human Immunodeficiency Virus; PCI, percutaneous coronary intervention; SD, standard deviation; STEMI, ST-elevation myocardial infarction.

**Table 2. Short and long-term hazards ratios by body mass index category**

|  | **30-Day HR (95% CI)** | **1-Year HR (95% CI)** | **5-Year HR (95% CI)** | **17-Year HR (95% CI)** |
| --- | --- | --- | --- | --- |
| **UNADJUSTED**  Underweight  Normal Weight  Overweight  Obese  Morbidly Obese | 1.00 | 1.00 | 1.00 | 1.00 |
| **ADJUSTED^a^**  Underweight  Normal Weight  Overweight  Obese  Morbidly Obese | 1.00 | 1.00 | 1.00 | 1.00 |

Abbreviations: AMI, acute myocardial infarction; CI, confidence interval; HR, hazard ratio.

^a^Adjusted for age, gender, race, ZIP code level median household income, cardiovascular risk factors (diabetes, hypertension, smoking, prior coronary events, and peripheral vascular disease), clinical presentation (Killip class, systolic blood pressure on admission, heart rate on admission, ST-elevation AMI, AMI location, and renal insufficiency), therapies received (percutaneous coronary intervention or coronary artery bypass grafting within 30 days of admission, fibrinolytic therapy during the index admission, and among those eligible, aspirin and beta-blockers on admission), cachexia-related factors (congestive heart failure, chronic obstructive pulmonary disease, cerebrovascular disease, chronic kidney disease, Human Immunodeficiency Virus, cancer, dementia, anemia, hypoalbuminemia), and measures of pre-hospital frailty (admission from a nursing home, decreased mobility, and urinary incontinence).

**Figure 1. Life expectancy after AMI by BMI category**

**Figure 2. Unadjusted years of potential life lost after AMI attributable to under or excess weight relative to normal weight patients**

**Figure 3. Adjusted years of potential life lost after AMI attributable to under or excess weight relative to normal weight patients**

**References**

1. Bucholz, E.M., et al., *Body mass index and mortality in acute myocardial infarction patients.* Am J Med, 2012. **125**(8): p. 796-803.

2. Das, S.R., et al., *Impact of body weight and extreme obesity on the presentation, treatment, and in-hospital outcomes of 50,149 patients with ST-Segment elevation myocardial infarction results from the NCDR (National Cardiovascular Data Registry).* J Am Coll Cardiol, 2011. **58**(25): p. 2642-50.

3. Mehta, L., et al., *Impact of body mass index on outcomes after percutaneous coronary intervention in patients with acute myocardial infarction.* Am J Cardiol, 2007. **99**(7): p. 906-10.

4. Nikolsky, E., et al., *Impact of body mass index on outcomes after primary angioplasty in acute myocardial infarction.* Am Heart J, 2006. **151**(1): p. 168-75.

5. Zeller, M., et al., *Relation between body mass index, waist circumference, and death after acute myocardial infarction.* Circulation, 2008. **118**(5): p. 482-90.

6. Aronson, D., et al., *The impact of body mass index on clinical outcomes after acute myocardial infarction.* Int J Cardiol, 2010. **145**(3): p. 476-80.

7. Buettner, H.J., et al., *The impact of obesity on mortality in UA/non-ST-segment elevation myocardial infarction.* Eur Heart J, 2007. **28**(14): p. 1694-701.

8. O'Brien, E.C., et al., *Association of body mass index and long-term outcomes in older patients with non-ST-segment-elevation myocardial infarction: results from the CRUSADE Registry.* Circ Cardiovasc Qual Outcomes, 2014. **7**(1): p. 102-9.

9. Wienbergen, H., et al., *Impact of the body mass index on occurrence and outcome of acute ST-elevation myocardial infarction.* Clin Res Cardiol, 2008. **97**(2): p. 83-8.

10. Herrmann, J., et al., *Body mass index and acute and long-term outcomes after acute myocardial infarction (from the Harmonizing Outcomes With Revascularization and Stents in Acute Myocardial Infarction Trial).* Am J Cardiol, 2014. **114**(1): p. 9-16.

11. Ikeda, N., et al., *Higher body mass index at the time of acute myocardial infarction is associated with a favorable long-term prognosis (8-year follow-up).* Heart Vessels, 2011. **26**(5): p. 495-501.

12. Kragelund, C., et al., *Impact of obesity on long-term prognosis following acute myocardial infarction.* Int J Cardiol, 2005. **98**(1): p. 123-31.

13. Habbu, A., N.M. Lakkis, and H. Dokainish, *The obesity paradox: fact or fiction?* Am J Cardiol, 2006. **98**(7): p. 944-8.

14. Lavie, C.J., A. De Schutter, and R.V. Milani, *Healthy obese versus unhealthy lean: the obesity paradox.* Nat Rev Endocrinol, 2015. **11**(1): p. 55-62.
